# Post‐Synthetic Mannich Chemistry on Metal‐Organic Frameworks: System‐Specific Reactivity and Functionality‐Triggered Dissolution

**DOI:** 10.1002/chem.201801419

**Published:** 2018-06-26

**Authors:** Harina Amer Hamzah, William J. Gee, Paul R. Raithby, Simon J. Teat, Mary F. Mahon, Andrew D. Burrows

**Affiliations:** ^1^ Department of Chemistry University of Bath Claverton Down Bath BA2 7AY United Kingdom; ^2^ School of Physical Sciences University of Kent Canterbury Kent CT2 7NZ United Kingdom; ^3^ Advanced Light Source Lawrence Berkeley National Laboratory 1 Cyclotron Road Berkeley CA 94720 USA

**Keywords:** Mannich reactions, mercury, metal-organic frameworks, post-synthetic modification, zirconium

## Abstract

The Mannich reaction of the zirconium MOF [Zr_6_O_4_(OH)_4_(bdc‐NH_2_)_6_] (UiO‐66‐NH_2_, bdc‐NH_2_=2‐amino‐1,4‐benzenedicarboxylate) with paraformaldehyde and pyrazole, imidazole or 2‐mercaptoimidazole led to post‐synthetic modification (PSM) through C−N bond formation. The reaction with imidazole (Him) goes to completion whereas those with pyrazole (Hpyz) and 2‐mercaptoimidazole (HimSH) give up to 41 and 36 % conversion, respectively. The BET surface areas for the Mannich products are reduced from that of UiO‐66‐NH_2_, but the compounds show enhanced selectivity for adsorption of CO_2_ over N_2_ at 273 K. The thiol‐containing MOFs adsorb mercury(II) ions from aqueous solution, removing up to 99 %. The Mannich reaction with pyrazole succeeds on [Zn_4_O(bdc‐NH_2_)_3_] (IRMOF‐3), but a similar reaction on [Zn_2_(bdc‐NH_2_)_2_(dabco)] (dabco=1,4‐diazabicyclo[2.2.2]octane) gave [Zn_3_(bdc‐NH_2_)_1.32_(bdc‐NHCH_2_pyz)_1.68_(dabco)]⋅2 C_7_H_8_ **5**, whereas the reaction with imidazole gave the expected PSM product. Compound **5** forms via a dissolution–recrystallisation process that is triggered by the “free” pyrazolate nitrogen atom competing with dabco for coordination to the zinc(II) centre. In contrast, the “free” nitrogen atom on the imidazolate is too far away to compete in this way. Mannich reactions on [In(OH)(bdc‐NH_2_)] (MIL‐68(In)‐NH_2_) stop after the first step, and the product was identified as [In(OH)(bdc‐NH_2_)_0.41_(bdc‐NHCH_2_OCH_3_)_0.30_(bdc‐N=CH_2_)_0.29_], with addition of the heterocycle prevented by steric interactions.

## Introduction

Metal‐organic frameworks (MOFs)^1^ are currently attracting considerable interest for their porosity properties, and applications as diverse as carbon capture,^2^ catalysis,^3^ drug delivery^4^ and chemical weapon detoxification.^5^ Much of this attention arises from the wide diversity of MOF structures, with variation of both the metal centres and organic linkers providing an essentially limitless number of possible materials. Of specific interest for many applications is the potential for forming functionalised MOFs,^6^ with particular functional groups appended to the pore walls. While such materials can sometimes be formed using a linker containing an appropriate substituent in the MOF synthesis, in practice many functional groups are intolerant to the synthetic conditions, or use of the functionalised linker in the synthesis gives rise to an unexpected product. Post‐synthetic modification (PSM)^7^ has emerged as a powerful tool for preparing such functionalised MOFs, and it is often the only way to place a particular substituent onto the pore walls of a MOF structure. A wide range of covalent post‐synthetic modification reactions have been developed over recent years, including conversion of primary amines into amides,^8^ isocyanates,^9^ ureas,^10^ azides,^11^ β‐amidoketones,^12^ secondary amines^13^ and diazonium salts,^14^ aldehydes into hydrazones,^15^ azides to triazoles,^16^ bromides to nitriles,^17^ as well as oxidation^18^ and reduction^19^ reactions. Despite this, there remains a need for new, versatile and synthetically‐straightforward methods that allow different functional groups to be incorporated into MOFs, regardless of their metal centres and framework structure.

The Mannich reaction, first reported over 100 years ago,^20^ involves the condensation of an amine with an aldehyde, normally formaldehyde, and a compound containing an active hydrogen.^21^ Originally, this latter compound was an enolisable carbonyl such as an ester or a ketone, but development of the reaction has seen other nucleophiles such as nitroalkanes,^22^ acetylenes^23^ and electron‐rich heterocycles, including pyrroles,^24^ furans^25^ and thiophenes,^26^ being employed as alternatives to carbonyl compounds. In this paper, we explore the post‐synthetic modification of the amino‐functionalised metal‐organic frameworks [Zr_6_O_4_(OH)_4_(bdc‐NH_2_)_6_] (UiO‐66‐NH_2_, bdc‐NH_2_=2‐amino‐1,4‐benzenedicarboxylate),^27^ [Zn_4_O(bdc‐NH_2_)_3_] (IRMOF‐3),^28^ [Zn_2_(bdc‐NH_2_)_2_(dabco)] (DMOF‐1‐NH_2_, dabco=1,4‐diazabicyclo[2.2.2]octane)^29^ and [In(OH)(bdc‐NH_2_)] (MIL‐68(In)‐NH_2_)^30^ using the Mannich reaction, employing pyrazole, imidazole and 2‐mercaptoimidazole as the nucleophiles. The products from these transformations were anticipated to have nitrogen and/or sulfur groups projecting into the pores and available for selective gas adsorption or metal ion uptake. In all cases presented herein, the Mannich reaction was carried out in two steps to prevent the nucleophile from reacting with formaldehyde, and no catalyst was required.

## Results and Discussion

### Mannich reactions on [Zr_6_O_4_(OH)_4_(bdc‐NH_2_)_6_], UiO‐66‐NH_2_

**Scheme 1:**
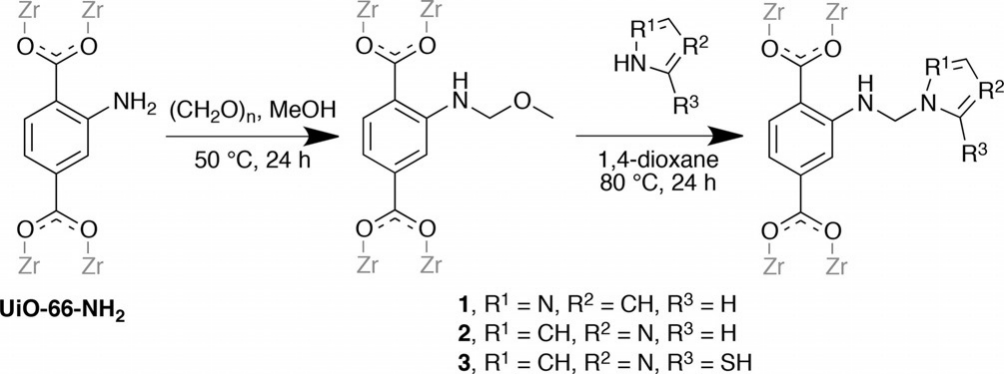
General procedure for the conversion of UiO‐66‐NH_2_ into azole‐functionalised MOFs **1**–**3**.

[Zr_6_O_4_(OH)_4_(bdc‐NH_2_)_6_], UiO‐66‐NH_2_, is an attractive PSM precursor due to the high chemical stability of the zirconium‐dicarboxylate framework, its high crystallinity and relatively large pore windows (≈6 Å),^31^ and the presence of the readily‐functionalised amino groups.^32^ Mannich reactions on UiO‐66‐NH_2_ were undertaken as shown in Scheme 1.

The first step involves the formation of methoxymethyl amine groups by the reaction with paraformaldehyde and MeOH at 50 °C. These methoxymethyl amine groups were subsequently converted into the final product by reaction with pyrazole, imidazole or 2‐mercaptoimidazole to give compounds **1**–**3**, respectively. All reactions proceeded without the need for a Lewis acid catalyst, which has the additional advantage of eliminating the work‐up associated with catalyst removal from the pores of the MOF and removes the possibility of pore blocking by the catalyst. The similarity between the PXRD patterns of UiO‐66‐NH_2_ and the PSM products **1**–**3** (Figure S2, S6 and S9) indicate that the original framework was maintained in all three cases.

The effectiveness of the PSM reactions in terms of the percentage conversion of amino groups into the Mannich products was gauged by ^1^H NMR spectroscopy. The ^1^H NMR spectra were obtained from MOF samples that were washed to remove unreacted reagents before digesting in NH_4_F/D_2_O with [D_6_]DMSO. For the reaction with pyrazole (Hpyz), the ^1^H NMR spectrum of **1** (Figure S3) shows a number of new signals in addition to those corresponding to the aromatic protons of the unmodified groups, present as D_2_bdc‐NH_2_ (*δ*=7.56d, 7.12s and 7.05d ppm). The aromatic protons of D_2_bdc‐NHCH_2_pyz were observed at *δ*=7.62d, 7.25s and 7.08d ppm, overlapping with the signals from D_2_bdc‐NH_2_ and others attributed to minor (<10 %) by‐products. The presence of the pyrazole ring on the digested framework of **1** was confirmed by the signals at *δ*=7.57 and 6.28 ppm. Attempts to remove the by‐products by thorough washing with a variety of solvents were unsuccessful, suggesting that these compounds are also derived from PSM reactions, with a double‐Mannich product the most likely. Formylated by‐products can be present in UiO‐66‐NH_2_, deriving from reaction with DMF during the MOF synthesis.^33^ NMR analysis on digested samples of UiO‐66‐NH_2_ showed no evidence for formylation, suggesting this is not the origin of the by‐products present in **1**.

By comparison of the integrals for the signals at *δ*=7.13 and 6.28 ppm, the percentage conversion from −NH_2_ into ‐NHCH_2_pyz groups was estimated to be 41 %. Ignoring the minor by‐products, this gives the formula for **1** as [Zr_6_O_4_(OH)_4_(bdc‐NH_2_)_3.54_(bdc‐NHCH_2_pyz)_2.46_]. Attempts to increase the degree of conversion by carrying out the reaction at a higher temperature or for a longer time period were unsuccessful, though it should be noted that higher conversion to the methoxymethyl amine in the first step might not be observable in the ^1^H NMR spectra of the digested product, given the likely reversion of any D_2_bdc‐NHCH_2_OMe to D_2_bdc‐NH_2_ under the acidic digestion conditions.

**Figure 1:**
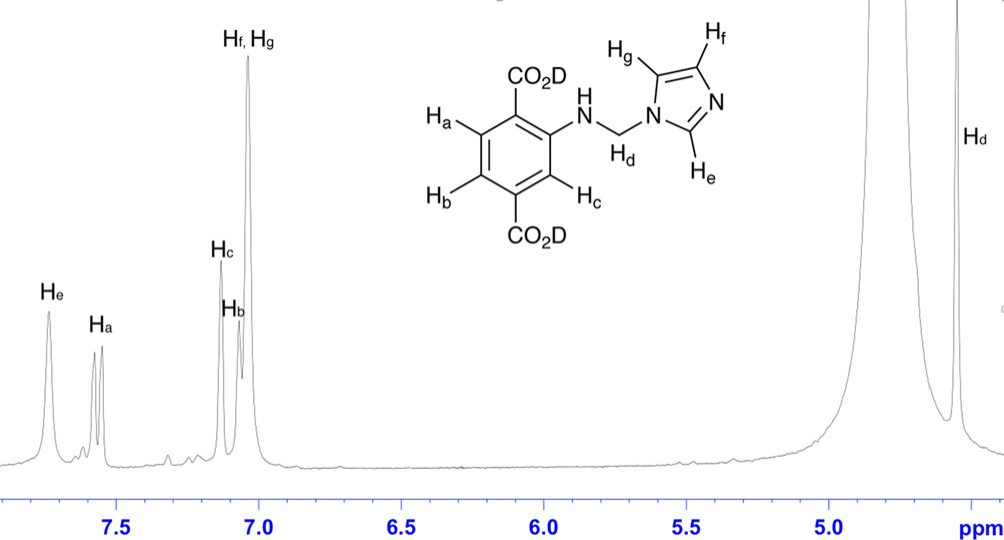
The ^1^H NMR spectrum of the product of the reaction between UiO‐66‐NH_2_, formaldehyde, methanol and imidazole, showing complete conversion to [Zr_6_O_4_(OH)_4_(bdc‐NHCH_2_im)_6_] **2**.

The Mannich reaction of UiO‐66‐NH_2_ with imidazole (Him) as the nucleophile was more successful than that with pyrazole, with the amino groups fully converted into ‐NHCH_2_im groups. This was confirmed by the disappearance of the signals which correspond to the aromatic protons of the starting MOF, UiO‐66‐NH_2_, in the ^1^H NMR spectrum of the digested product. Instead, new signals at *δ*=7.56d, 7.14s and 7.07d ppm were observed (Figure 1), corresponding to the protons from the benzene ring of D_2_bdc‐NHCH_2_im. Furthermore, the presence of the imidazole ring can be confirmed by the presence of two singlets in the aromatic region (*δ*=7.75 and 7.03 ppm). The signal at *δ*=7.03 ppm corresponds to two chemically similar but non‐identical protons from the imidazole ring, and this overlaps with the doublet from one of the aryl protons, whereas the singlet at *δ*=7.75 ppm arises from the remaining proton peak of the imidazole ring. The signal attributed to the methylene protons can be seen at *δ*=4.56 ppm, close to the broad HDO peak resulting from the digestion solvent. The chemical formula of this PSM product is [Zr_6_O_4_(OH)_4_(bdc‐NHCH_2_im)_6_] **2**.

In contrast to the complete conversion observed for **2**, the comparable Mannich reaction with 2‐mercaptoimidazole (HimSH) as the nucleophile gave only partial conversion. The ^1^H NMR spectrum (Figure S10) of the digested product **3** shows the presence of new peaks in addition to the aromatic proton peaks which correspond to the starting MOF, UiO‐66‐NH_2_. The signals attributed to the aromatic protons of D_2_bdc‐NHCH_2_imSH are observed at *δ*=7.68d, 7.26s and 7.08d ppm, respectively, although these peaks overlap with others from minor by‐products. The presence of new peaks at *δ*=6.98 and 6.76 ppm, from the imidazole ring, indicates that the 2‐mercaptoimidazole ring was successfully grafted onto the MOF framework.

The percentage conversion from ‐NH_2_ into ‐NHCH_2_imSH was calculated as approximately 36 % by comparing the integrals for the signals at *δ*=7.16 and 6.76 ppm. Ignoring minor by‐products, this gives a formula for **3** of [Zr_6_O_4_(OH)_4_(bdc‐NH_2_)_3.84_(bdc‐NHCH_2_imSH)_2.16_].

For **1**–**3**, further evidence for successful PSM came from the ESI mass spectra of the digested products. The negative ion ESI mass spectra of digested **1** and **3** confirmed the presence of the deprotonated anions of H_2_bdc‐NHCH_2_pyz and H_2_bdc‐NHCH_2_imSH at *m*/*z=*260.0664 (predicted [*M*−H]^−^=260.0671) and *m*/*z=*292.0400 (predicted [*M*−H]^−^=292.0392), respectively. In both cases a peak was also observed for H_2_bdc‐NH_2_ (*m*/*z=*180.0308, predicted [*M*−H]^−^=180.0297). Digested **2** gave better results in the positive ion rather than the negative ion ESI mass spectrum, with the protonated cation of H_2_bdc‐NHCH_2_im observed at *m*/*z=*262.0824 (predicted [*M*+H]^+^=262.0828).

The percentage conversions for the PSM reactions generating **1**–**3** are summarised in Table 1. The differences in degree of conversion can be related to the nucleophile strength. Imidazole is a stronger nucleophile than pyrazole due to its higher basicity, and is therefore more susceptible to nucleophilic substitution with −NHCH_2_OCH_3_, leading to a higher conversion. The steric demands of the nucleophile also have some influence on the extent of the reaction, with the lowest conversion achieved in the case of 2‐mercaptoimidazole, the largest of the nucleophiles employed. This can be rationalised by the more restricted diffusion of 2‐mercaptoimidazole within the pores of the MOF.

**Table 1:** The effect of the nucleophile on the degree of conversion observed in the Mannich reaction. The reactions were carried out using the conditions shown in Scheme 1.

Compound	Nucleophile	% Conversion
**1**	pyrazole	41
**2**	imidazole	100
**3**	2‐mercaptoimidazole	36

The thiol substituent in **3** was anticipated to be able to coordinate to soft metal centres such as mercury(II). In order to probe the effect of different ‐NHCH_2_imSH loadings on Hg^II^ uptake, a second thiol‐containing MOF was prepared, using the same conditions as for **3**, but with the temperature for the second step reduced from 80 to 50 °C. It was anticipated that the lower temperature during the second step would lead to a lower conversion to the ‐NHCH_2_imSH group.

The ^1^H NMR spectrum (Figure S13) of the digested product formed under these conditions, **3 a**, showed the presence of the modified group (‐NHCH_2_imSH), though present in a lower relative concentration than in **3**. The percentage conversion from ‐NH_2_ into ‐NHCH_2_imSH groups was estimated as 21 %, giving a formula for **3 a** of [Zr_6_O_4_(OH)_4_(bdc‐NH_2_)_4.74_(bdc‐NHCH_2_imSH)_1.26_]. This confirms that the reaction temperature has a significant impact on the degree of modification, with a lower temperature leading to lower conversion.

The TGA profiles of the PSM products **1**–**3** and **3 a** exhibit similar features to that for UiO‐66‐NH_2_ (Figure S14). There is an initial mass loss (up to 110 °C) corresponding to removal of 1,4‐dioxane from the pores. A small, gradual mass loss, observed in the range 110–470 °C, is attributed to the loss of residual solvent in the pores and/or the dehydroxylation of the Zr_6_O_4_(OH)_4_ nodes.^34^ The final mass loss, beginning at 470 °C, is due to the decomposition of the framework. Based on the TGA profiles, **1** has 4.0, **2** has 3.0, **3** has 5.0, **3 a** has 5.5 and UiO‐66‐NH_2_ has 7.0 molecules of 1,4‐dioxane per Zr_6_O_4_(OH)_4_ unit in the unactivated MOFs. This shows that the amount of 1,4‐dioxane in the pores decreases as the degree of post‐synthetic modification increases. This is unsurprising, since the greater the degree of conversion, the lower the residual space available to accommodate guest solvent molecules.

**Figure 2:**
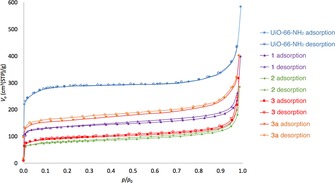
N_2_ sorption isotherms for compounds **1**–**3** and **3 a** at 77 K, in comparison to that for UiO‐66‐NH_2_.

The BET surface areas of **1**–**3** and **3 a** were determined based on their N_2_ adsorption isotherms at 77 K (Figure 2). The compounds were activated using the conventional activation temperature for UiO‐66 and its derivatives (120 °C for 12 h), and the BET surface area for UiO‐66‐NH_2_ obtained in this work (*S*_BET_=1041 m^2^ g^−1^) is similar to previously reported values.^35^ All PSM products exhibit type I isotherms, indicative of microporous materials, and have lower BET surface areas than UiO‐66‐NH_2_, with *S*_BET_ values of 528 m^2^ g^−1^ for **1**, 290 m^2^ g^−1^ for **2**, 352 m^2^ g^−1^ for **3** and 608 m^2^ g^−1^ for **3 a**. BET surface areas are governed by the degree of conversion and the size of the modified groups. In general, the BET surface area reduces as the percentage conversion increases and **2**, with complete conversion, has the lowest surface area. The presence of larger pendant groups in the pores also leads to lower BET surface areas, with the value for **3** less than that for **1**, despite **1** possessing a higher degree of modification.

The CO_2_ adsorption isotherms of the PSM products were measured at 273 K (Figure S15) to assess the influence of the modified groups on the CO_2_ uptake capacities. All PSM products show lower CO_2_ uptake capacities than UiO‐66‐NH_2_, attributable to the reduction in pore volume and the lower percentage of −NH_2_ groups in the pores. Of the PSM products, **1** shows the highest CO_2_ uptake which is probably due to the favourable interactions of CO_2_ molecules with the nitrogen atom in the pyrazole ring. Compound **2** shows a lower CO_2_ uptake than **1**, despite having higher percentage of heterocycles in the pores, which is consistent with the lower BET surface area, itself a consequence of the high degree of modification. Compounds **3** and **3 a** show the lowest CO_2_ uptake capacities at 1 bar and this may be due to pore blocking caused by higher steric hindrance of the modified groups. Nonetheless, the proportion of thiol groups in the pores has little impact on the CO_2_ uptake capacities, as evidenced by the relatively small difference in CO_2_ uptake between **3** and **3 a**. The modified MOFs show enhanced CO_2_/N_2_ selectivity over UiO‐66‐NH_2_, though this is largely a consequence of their low N_2_ uptake at 273 K.

The thiol‐containing PSM products, [Zr_6_O_4_(OH)_4_(bdc‐NH_2_)_3.84_(bdc‐NHCH_2_imSH)_2.16_] **3** and [Zr_6_O_4_(OH)_4_(bdc‐NH_2_)_4.74_(bdc‐NHCH_2_imSH)_1.26_] **3 a** were also investigated for their ability to remove mercury(II) from aqueous solutions. The Hg^II^ uptake experiments were carried out by immersing the MOF samples in an aqueous solution of HgCl_2_ (100 ppm) and stirring the solution for 12 h at ambient temperature. The Hg^II^‐treated MOFs were isolated by centrifugation and atomic emission spectroscopy (AES) was used to quantify the residual Hg^II^ concentration in the supernatant.

Mercury uptake capacities were calculated using [Equation (1)] where C_i_ and C_e_ represent the initial and equilibrium Hg^II^ concentrations, respectively. In addition to PSM products **3** and **3 a**, the Hg^II^ uptake capacities of the unmodified MOFs, UiO‐66 and UiO‐66‐NH_2_, were investigated for comparison, with the results presented in Table 2.

(1) $$\mathrm{Hg}\left(\mathrm{II}\right)\;\mathrm{uptake}\;\left(\%\right)=\frac{\left(C_{i}{-C}_{e}\right)}{C_{i}}\times 100$$

**Table 2:** The Hg^II^ uptake capacities of UiO‐66, UiO‐66‐NH_2_ and compounds **3** and **3 a**.

Compound	C_Hg(II)_ prior to MOF treatment, C_i_ [ppm]	C_Hg(II)_ after MOF treatment, C_e_ [ppm]	Hg[II] uptake [%]
UiO‐66	100	89	11
UiO‐66‐NH_2_	100	77	23
**3**	100	50	50
**3 a**	100	1	99

The post‐synthetic grafting of thiol groups in the pores of UiO‐66 proved to be beneficial for Hg^II^ absorption, as the uptake capacities were significantly increased for **3** and **3 a** over the unmodified MOFs. Perhaps surprisingly, the highest Hg^II^ uptake was observed for **3 a**, despite **3** having a higher loading of thiol groups in the pores. This reflects the lower porosity of **3**, which is likely to lead to some of the thiols being unavailable to interact with the Hg^II^ ions. The Hg^II^ uptake in **3 a** is comparable to that reported for the previously reported derivative UiO‐66‐(SH)_2_,^36^ which is one of the highest reported for a MOF, demonstrating the potential of **3 a** for mercury removal. PXRD (Figure S17) confirmed that **3 a** retains its crystallinity on treatment with HgCl_2_ (aq).

### Mannich reactions on [Zn_4_O(bdc‐NH_2_)_3_], IRMOF‐3

IRMOF‐3 contains large channels (≈9.6 Å) and there is considerable precedence for the post‐synthetic modification of the amino groups that protrude into its pores.^28^ As IRMOF‐3 has a low stability towards moisture and alcohols,^37^ toluene was selected as the optimum solvent for the Mannich reaction.

**Scheme 2:**
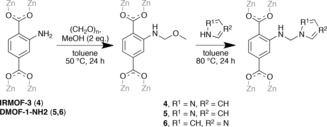
Mannich reactions on IRMOF‐3 and DMOF‐1‐NH_2_.

To demonstrate the applicability of Mannich reaction on IRMOF‐3, the PSM reaction with pyrazole was carried out using the reaction conditions outlined in Scheme 2.

The effectiveness of the PSM reaction was gauged by ^1^H NMR spectroscopy on the DCl/D_2_O‐digested product **4** (Figure S19). In addition to the signals corresponding to the aryl protons of D_2_bdc‐NH_2_, new features attributed to the aryl protons of the modified product were observed at *δ*=7.89d, 7.46d and 7.20dd ppm. The successful incorporation of the‐NHCH_2_pyz groups could also be evidenced by the emergence of new signals at *δ*=7.85d, 7.72d and 6.25dd ppm, corresponding to the protons of the pyrazole ring. The peak attributed to the methylene protons was located at *δ*=5.68 ppm. The degree of conversion was calculated by comparing the integrals at *δ*=7.46 and 7.42 ppm and found to be 75 %, giving the formula for **4** as [Zn_4_O(bdc‐NH_2_)_0.75_(bdc‐NHCH_2_pyz)_2.25_].

The negative ion ESI mass spectrum of the digested product **4** confirms the presence of the deprotonated anions of H_2_bdc‐NHCH_2_pyz and H_2_bdc‐NH_2_ at *m*/*z=*260.0669 (predicted [*M*−H]^−^=260.0671) and *m*/*z=*180.0308 (predicted [*M*−H]^−^=180.0297), respectively. The PXRD pattern of **4** (Figure S18) shows the similarities in peak positions with the starting MOF, IRMOF‐3, indicating that the bulk framework structure remained unchanged upon PSM. Nonetheless, a degree of degradation was observed, as evidenced by the broadening of peaks and reduced intensities. The presence of stoichiometric MeOH in the first step and as a side product in the second step may cause some crystal degradation. Attempts to analyse **4** by single crystal X‐ray crystallography were unsuccessful due to poor diffracting power of the sample.

### Mannich reactions on [Zn_2_(bdc‐NH_2_)_2_(dabco)], DMOF‐1‐NH_2_

[Zn_2_(bdc‐NH_2_)_2_(dabco)], DMOF‐1‐NH_2_, is a flexible MOF which consists of Zn_2_(dicarboxylate)_2_ sheets that are linked by dabco pillars into a three‐dimensional network.^29^ MOFs in this series are able to undergo transitions from narrow rhomboidal pores to open, square pores, and this can be influenced by solvent or substituent.^38^ Toluene was selected as a solvent for post‐synthetic Mannich reactions on DMOF‐1‐NH_2_ due to it having little effect on the pore geometry and it not unduly affecting the crystallinity.

To demonstrate the applicability of the Mannich reaction on DMOF‐1‐NH_2_, the reaction was carried out using the same conditions as outlined for IRMOF‐3 in Scheme 2. The ^1^H NMR spectrum of the digested product **5** (Figure S24) shows the presence of aromatic protons attributed to D_2_bdc‐NH_2_ (*δ*=7.82, 7.48 and 7.13 ppm) and D_2_bdc‐NHCH_2_pyz (*δ*=7.89, 7.49 and 7.20 ppm). The peaks at *δ*=7.13 and 7.20 ppm overlap with the signals from the aryl protons of residual toluene solvent. The protons of the pyrazole ring are located at *δ*=7.85 7.72, and 6.25 ppm. The peak attributed to the α−CH_2_ protons is observed at *δ*=5.68 ppm although there is some overlap between this peak and that for HDO, present from the digestion mixture. Comparing the integrals of the protons at *δ*=7.48–7.49 ppm and *δ*=6.25 ppm, the percentage conversion of amino into ‐NHCH_2_pyz groups was calculated to be 56 %.

The negative ion ESI mass spectrum of the digested product **5** confirmed the presence of the deprotonated anions of H_2_bdc‐NHCH_2_pyz and H_2_bdc‐NH_2_ at *m*/*z=*260.0662 (predicted [*M*−H]^−^=260.0671) and *m*/*z=*180.0364 (predicted [*M*−H]^−^=180.0375), respectively. The disappearance of ‐NH_2_ stretching bands (3287 and 3457 cm^−1^) of DMOF‐1‐NH_2_ in the FTIR spectrum of **5** (Figure S26) indicates the successful conversion of primary into secondary amine.

The PXRD pattern of **5** is completely different to that of DMOF‐1‐NH_2_ (Figure S22), revealing a significant structural difference between the two materials. Indeed, the PXRD pattern of **5** does not match any of the PXRD patterns reported in the literature for DMOF‐1 type materials. Inspection of **5** under an optical microscope revealed the presence of small colourless crystals and the absence of brown block crystals, characteristic of DMOF‐1‐NH_2_ and its derivatives. This observation suggests that DMOF‐1‐NH_2_ has undergone a complete structural change upon reaction.

**Figure 3:**
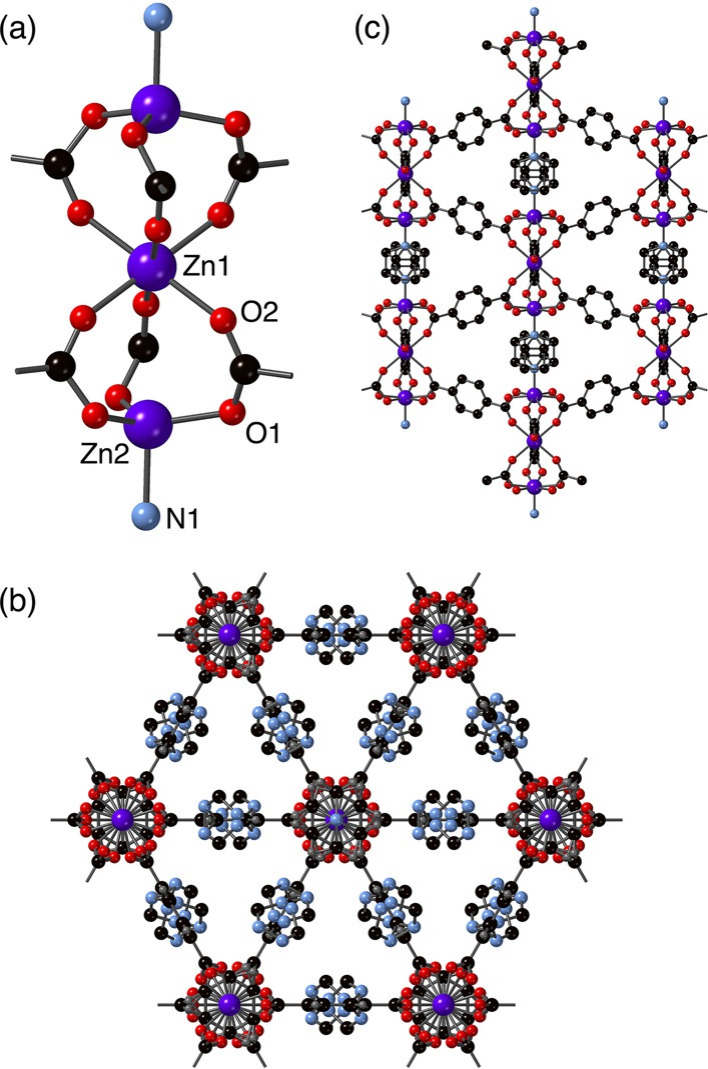
The structure of [Zn_3_(bdc‐NH_2_)_1.32_(bdc‐NHCH_2_pyz)_1.68_(dabco)]⋅2 C_7_H_8_ **5**, showing (a) the Zn_3_(O_2_CR)_6_ SBU, and the gross structure of the framework viewed (b) along and (c) perpendicular to the *c*‐axis. In (c), the hydrogen atoms and tag groups are omitted for clarity.

The crystal structure of **5** was successfully elucidated by single crystal X‐ray crystallography and is shown in Figure 3. The compound crystallises in the trigonal space group *R*‐3m, and the asymmetric unit (Figure S44) contains one quarter of a zinc atom (Zn1 and Zn2 have 8.333 % and 16.667 % occupancy, respectively), one twelfth of a dabco ligand and one quarter of a ligand which is comprised of bdc‐NH_2_ and bdc‐NHCH_2_pyz, disordered in a 34:56 ratio.

Attempts to accurately determine the structural void volume via the PLATON SQUEEZE algorithm were hampered by pendant group site‐occupancies, disorder and the smearing of electron density. The TGA of **5** indicates a mass loss that corresponds to two toluene molecules for every three zinc centres present, and this provides a formulation of **5** as [Zn_3_(bdc‐NH_2_)_1.32_(bdc‐NHCH_2_pyz)_1.68_(dabco)]⋅2 C_7_H_8_.

Overall, the SBU in **5** contains three zinc centres, one 6‐coordinate and two 4‐coordinate (Figure 3 a). The Zn1 metal centre is in a distorted octahedral coordination environment, and is coordinated to six O2 donor atoms, each from a different carboxylate group. In contrast, Zn2 exhibits a distorted tetrahedral coordination geometry, being coordinated to three O1 donor atoms from different carboxylate groups and to the nitrogen atom N1 of the dabco ligand.

The Zn_3_(O_2_CR)_6_ SBUs are pillared by the dabco ligands along the *c* axis and these pillars are linked in the *ab* plane by the substituted bdc linkers to form a three‐dimensional network containing infinite one‐dimensional triangular channels (Figure 3 b,c). The crystallographically located atoms in the modified groups protrude into the channels. The Zn_3_(O_2_CR)_6_ SBU exhibited by **5** has previously been observed in other MOF systems. For example, a three‐dimensional MOF, [Zn_3_(bpdc)_3_(bpy)], (bpd=4,4′‐biphenyl dicarboxylate, bpy=4,4′‐bipyridine) prepared by Li and co‐workers,^39^ contains zinc(II) metal centres which exhibit the same coordination geometries as those in **5**.

**Figure 4:**
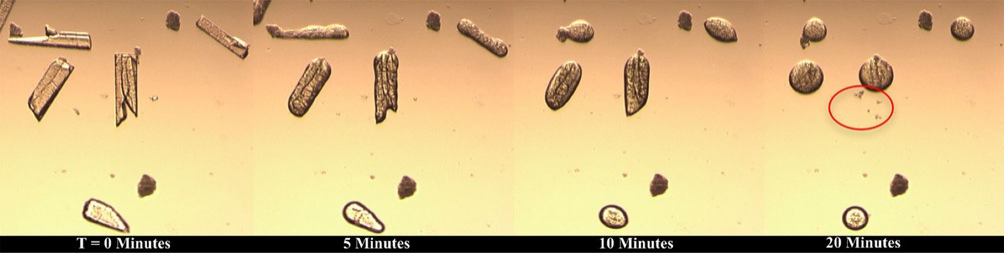
Four frames at *t=*0, 5, 10 and 20 min of the dissolution of DMOF‐1‐NHCH_2_OCH_3_ crystals in the presence of a solution of toluene and pyrazole. Small crystals of **5** can be seen starting to form near the centre of the final frame.

In order to investigate the cause of the structural transformation from DMOF‐1‐NH_2_ into **5**, a series of control studies were carried out. No structural change was observed when DMOF‐1‐NH_2_ crystals were heated in toluene, or when the crystals were treated separately with paraformaldehyde, MeOH or pyrazole (Figure S27). This suggested that the formation of the methoxymethyl amine intermediate DMOF‐1‐NHCH_2_OCH_3_ in the first step was unproblematic, but that the structural transformation occurred in the second step of the Mannich reaction. In order to confirm this, the reaction of DMOF‐1‐NHCH_2_OCH_3_ with pyrazole was monitored under an optical microscope equipped with a camera. The reaction conditions were modified in order to be able to view the reaction in this way. In particular, DMOF‐1‐NHCH_2_OCH_3_ crystals were dispersed on a microscope slide containing a solution of pyrazole in toluene at room temperature. After five minutes, the crystals began to dissolve, with complete dissolution observed after 40 minutes. A new phase, corresponding to the crystals of **5**, was first observed after approximately twenty minutes (Figure 4), confirming that **5** is produced in a dissolution‐re‐precipitation process.

**Figure 5:**
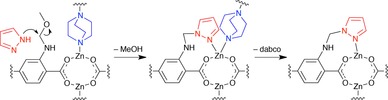
Proposed mechanism for the dissociation of the DMOF‐1 structure on reaction with pyrazole.

Although it is not possible to provide a definitive mechanism for the dissociation of DMOF‐1‐NHCH_2_OCH_3_, a proposed reaction mechanism that leads to the dissociation of the SBUs is shown in Figure 5.

After the first step of the Mannich reaction, the methoxymethyl amine species is localised in close proximity to the bridging dabco ligands. Upon addition of pyrazole, a facile reaction displacing methanol can occur to yield the ‐NHCH_2_pyz group, which is aligned in such a way as to compete in an intramolecular manner with dabco for coordination to the Zn^II^ metal centre. Displacement of dabco would break the three‐dimensional network of the DMOF‐1 framework, leading to rapid delamination, and ultimately triggering framework dissolution. Notably, in the crystal structure of **5**, the ‐NHCH_2_pyz group is directed away from the dabco ligand (Figure S43), so is unable to compete with it for coordination. Moreover, a diaza‐[18]‐crown‐6 ligand functionalised with pendant pyrazole groups using a Mannich reaction also exhibited fragmentation behaviour in the presence of transition metals,^40^ leading further credence to this hypothesis.

It should be noted that PXRD patterns for bulk samples of **5** show the presence of more than one phase, so the degree of occupancy of the pores by toluene in the crystal structure is an estimate, and although the ratio of linkers in the ^1^H NMR spectra are consistent between samples, this too may have been different in the crystal analysed crystallographically.

The Mannich reaction of DMOF‐1‐NH_2_ with imidazole as the nucleophile was carried out using the same conditions as with pyrazole (Scheme 2). The ^1^H NMR spectrum of the digested product **6** (Figure S30) shows aromatic protons from D_2_bdc‐NH_2_ and D_2_bdc‐NHCH_2_im, and from the integrals the percentage conversion of amino into ‐NHCH_2_im groups was calculated to be 65 %, giving a formula for **6** as [Zn_2_(bdc‐NH_2_)_0.7_(bdc‐NHCH_2_im)_1.3_(dabco)]. The negative ion ESI mass spectrum of the acid‐digested product **6** confirmed the presence of the deprotonated anions of H_2_bdc‐NHCH_2_im and H_2_bdc‐NH_2_ at *m*/*z=*260.0661 (predicted [*M*−H]^−^=260.0671) and *m*/*z=*180.0339 (predicted [*M*−H]^−^=180.0297).

The PXRD pattern of **6** and the starting MOF, DMOF‐1‐NH_2_ closely match one another (Figure S29), demonstrated that PSM does not affect the gross structure or the crystallinity of the product. Furthermore, visual inspection of **6** confirmed the presence of only brown block crystals and the absence of new phases. Attempts to analyse **6** crystallographically were hampered by crystal twinning. Nonetheless, a screening experiment suggested that there were similarities in the unit cell parameters of **6** (*a=*15.2955(17) Å, *b=*15.2860(15) Å, *c=*19.207(2) Å) and those of DMOF‐1 (*a=*15.063(2) Å, *c=*19.247(5) Å).

Based on these results, it is clear that framework dissolution does not occur when imidazole is used as a nucleophile. It is believed that substituting pyrazole by imidazole prevents the dissolution of DMOF‐1‐NHCH_2_OCH_3_, by eliminating the possibility of coordinative competition with dabco. The “free” nitrogen atom in imidazole is positioned beyond the coordination sphere of the zinc(II) centre and, as a consequence, the process shown in Figure 5 is unable to occur.

The Mannich reaction of DMOF‐1‐NH_2_ with 2‐mercaptoimidazole as the nucleophile was attempted using the same conditions as in the reaction with imidazole. However, the ^1^H NMR spectrum of the digested solid showed only signals corresponding to the aryl protons of DMOF‐1‐NH_2_ (Figure S37), indicating that the inclusion of 2‐mercaptoimidazole onto this MOF framework was unsuccessful. The PXRD pattern (Figure S36) is similar to that for DMOF‐1‐NH_2_, implying that the framework was retained throughout the experiment. The unsuccessful grafting of 2‐mercaptoimidazole onto the MOF framework is likely to be due to its larger size than imidazole, which makes it too big to pass through the pore windows (2‐mercaptoimidazole: 8.4×6.6 Å. DMOF‐1‐NH_2_ channels: 5.3×4.8 Å).

### Mannich reactions on [In(OH)(bdc‐NH_2_)], MIL‐68(In)‐NH_2_

[In(OH)(bdc‐NH_2_)]_,_ MIL‐68(In)‐NH_2_, is a three‐dimensional MOF that is constructed from chains of InO_4_(OH)_2_ octahedral units that are linked together by bdc‐NH_2_ ligands to form triangular (≈6 Å) and hexagonal (≈16 Å) one‐dimensional channels. In MIL‐68(In)‐NH_2_, the amino groups are oriented towards the InO_4_(OH)_2_ octahedral chains rather than projecting into the pores. However, this has not prevented successful tandem post‐synthetic modifications involving formation of the azide and subsequent click reactions from being carried out,^11^ so presumably some flexibility is possible to accommodate the bulkier, modified groups.

MIL‐68(In)‐NH_2_ was prepared using an analogous synthesis to that for MIL‐68(In), originally reported by Loiseau and co‐workers.^30^ In a typical PSM procedure, MIL‐68(In)‐NH_2_ crystals were treated with paraformaldehyde and MeOH at 50 °C for 24 h. In this reaction, MeOH was used as a reactant as well as a solvent, as MIL‐68(In) is stable towards alcohols, thus eliminating the need to use a different solvent. The intermediate product was then washed with 1,4‐dioxane and treated with pyrazole at 80 °C for 24 h, before quenching the reaction by washing the sample with fresh 1,4‐dioxane.

The ^1^H NMR spectrum of the digested PSM product **7** (Figure S39) was obtained by digesting the MOF in a basic aqueous solution (NaOD/D_2_O). In addition to the signals corresponding to the aromatic protons of Dbdc‐NH_2_^−^, two new sets of signals were observed in the downfield region of the spectrum. However, the absence of peaks attributed to the protons of the pyrazole ring indicated that the PSM reaction did not afford the expected pyrazole‐containing product. The signals at *δ*=7.73d, 7.36s and 7.15d ppm are believed to be due to the aryl protons from the intermediate MOF, MIL‐68(In)‐NHCH_2_OCH_3_, observed as Dbdc‐NHCH_2_OCH_3_^−^ in the NMR spectrum. The peaks attributed to the methylene protons and methyl terminus of Dbdc‐NHCH_2_OCH_3_^−^ are located at *δ*=4.87 and 4.76 ppm, respectively, although these are partly obscured by the peak from HDO, present from the digestion solvent. The other signals, at *δ*=7.68d, 7.43s and 7.18d ppm, are believed to be from the imine Dbdc‐N=CH_2_^−^, with mass spectrometry providing support for this (vide infra).

**Scheme 3:**
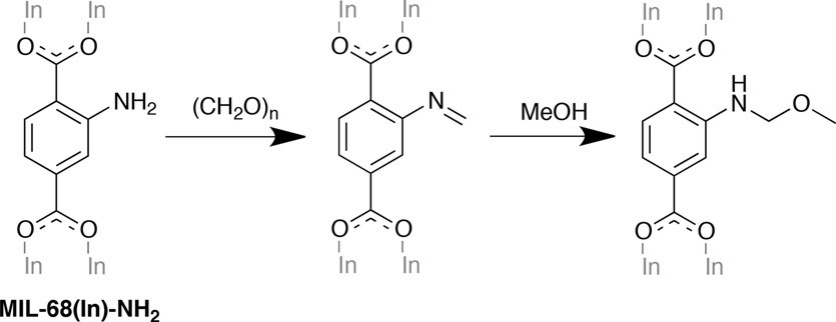
The first step of the Mannich reaction carried out on MIL‐68(In)‐NH_2_.

In order to confirm that the observed products do not require the presence of pyrazole, the reaction mixture was analysed prior to its addition. The first step of the Mannich reaction is depicted in Scheme 3, broken down into two stages. As anticipated, the ^1^H NMR spectrum of the digested product (Figure S40) illustrates a high similarity with that for **7**, with only small differences in the relative proportions of the two products.

This finding validates the hypothesis that **7** contains unreacted −NH_2_ groups, as well as imine and methoxymethyl amine species. The presence of the imine could be due to incomplete reaction with methanol or, alternatively, from the partial hydrolysis of D_2_bdc‐NHCH_2_OCH_3_ in the digestion medium. Given that D_2_bdc‐NHCH_2_OCH_3_ appears to be stable under the digestion conditions, the most reasonable formulation for **7** includes both substituents, and can be represented by the formula [In(OH)(bdc‐NH_2_)_0.41_(bdc‐NHCH_2_OCH_3_)_0.30_(bdc‐N=CH_2_)_0.29_].

The negative ion ESI mass spectrum of the base‐digested product **7** (Figure S41) confirms the presence of the deprotonated anions of H_2_bdc‐NHCH_2_OCH_3_ ([*M*−H]^−^=224.0560, predicted 224.0559), H_2_bdc‐N=CH_2_ ([*M*−H]^−^=192.0310, predicted 192.0297) and H_2_bdc‐NH_2_ ([*M*−H]^−^=180.0315, predicted 180.0297), and provides good evidence for the identity of the tag groups in the products. The PXRD pattern of **7** is similar to that of MIL‐68(In)‐NH_2_ (Figure S38), indicating that framework integrity is maintained and the PSM reaction did not alter the crystallinity of the product.

Crystals were grown from dioxane, and the crystal structure of **7**⋅0.8 dioxane was successfully elucidated by single crystal X‐ray crystallography. The asymmetric unit (Figure S46) consists of two indium(III) centres with site occupancies of 0.5 and 0.25 for In1 and In2, respectively, one half and one quarter of a dicarboxylate ligand and two OH ligands (based on O1 and O5) with combined site occupancies of 0.75. Finally, there was evidence for some diffuse solvent present in the framework which was modelled as four‐fifths of a dioxane molecule per indium centre based on TGA evidence.

**Figure 6:**
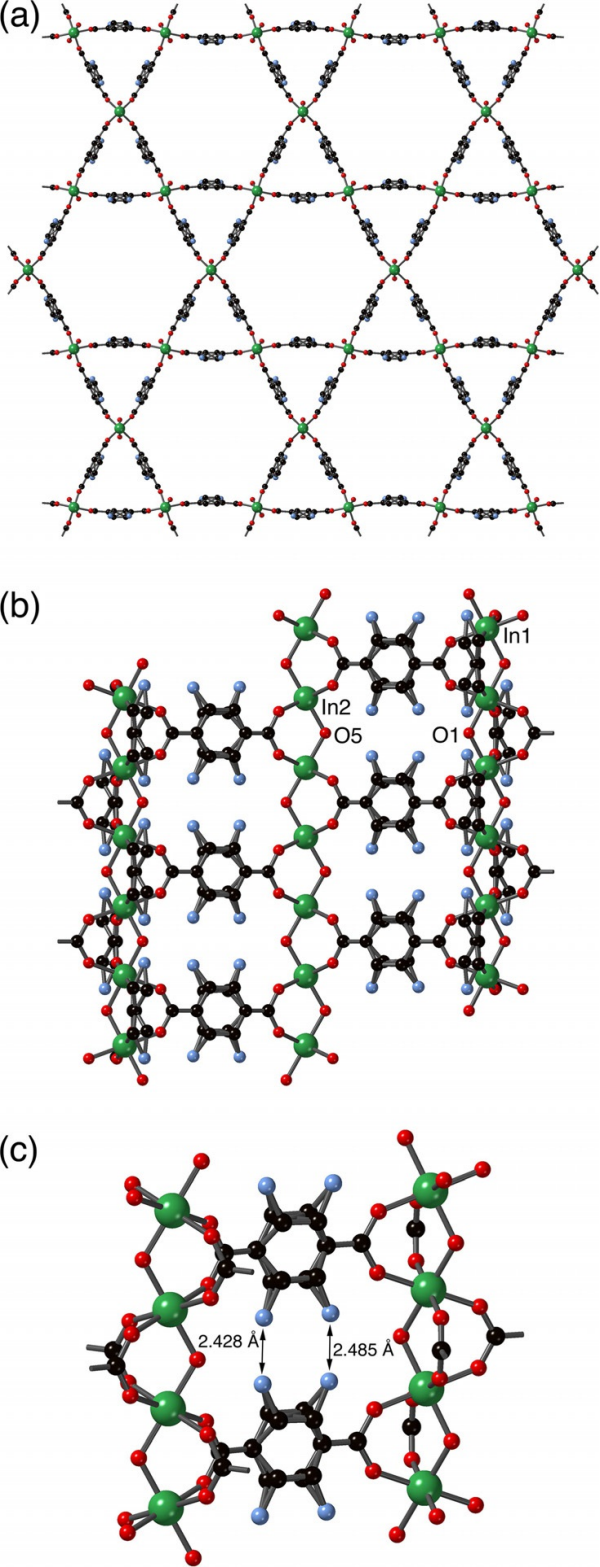
The structure of **7**⋅0.8dioxane, showing (a) the kagome lattice adopted by MIL‐68 analogues, (b) the interlinking of the In(OH)(O_2_CR)_2_ chains, and (c) the close proximity of the partial occupancy nitrogen atoms, illustrating the steric barrier to reaction of the methoxymethyl amine with pyrazole.

As can be seen in Figure 6 a,b, the overall framework topology has not changed significantly during the reaction from that of MIL‐68(In)‐NH_2_, in agreement with the PXRD data. Although there is some evidence for the nitrogen atoms of the tag groups, disordered over several positions, further evidence for the nature of the substituents was unavailable.

The most notable insight from the crystal structure of **7** is the short distance between the nitrogen atoms on neighbouring benzene rings (Figure 6 c). Although these atoms have only partial occupancy, this proximity illustrates the difficulties involved in placing a large substituent on one of these atoms. This provides justification for the argument that the second step of the Mannich reaction is disfavoured in this case on steric grounds.

## Conclusion

The results presented herein demonstrate a previously unreported post‐synthetic modification process on MOFs, whereby catalyst‐free Mannich reactions were used to convert the primary amines of UiO‐66‐NH_2_, IRMOF‐3, DMOF‐1‐NH_2_ and MIL‐68(In)‐NH_2_ into a range of azole‐functionalised MOFs with conversions of up to 100 %. It did not prove possible to prepare the azole‐functionalised acids using similar procedures, and the instability of N−CH_2_−N linkages to hydrolysis is well established.^41^ Hence post‐synthetic modification provides the only method to prepare these functionalised MOFs.

With regards to the PSM reactions on UiO‐66‐NH_2_, the degree of conversion from ‐NH_2_ into ‐NHR (R=CH_2_pyz, CH_2_im and CH_2_imSH) depends on the strength and size of the nucleophiles. Complete conversion was achieved with the strongest nucleophile (imidazole) whereas a lower conversion (41 %) was obtained with the isosteric weaker nucleophile, pyrazole. The use of a larger nucleophile, 2‐mercaptoimidazole, led to the lowest conversion (36 %) and this is most likely due to the restricted diffusion of the nucleophile within the pores of UiO‐66‐NH_2_. The modified MOFs have lower BET surface areas than UiO‐66‐NH_2_, but show enhanced selectivity for CO_2_ over N_2_. In addition, the thiol‐containing products show excellent uptake of mercury(II) from aqueous solutions.

With regard to the PSM reaction on IRMOF‐3, 75 % conversion of ‐NH_2_ into ‐NHCH_2_pyz was achieved whilst using pyrazole as a nucleophile. However, the successful PSM reaction comes at a cost of decreased product crystallinity as evidenced by the broadening of peaks and reduction in peak intensities in the PXRD pattern of the PSM product.

The Mannich reaction on DMOF‐1‐NH_2_, using pyrazole as the nucleophile, unexpectedly afforded [Zn_3_(bdc‐NH_2_)_1.32_(bdc‐NHCH_2_pyz)_1.68_(dabco)]⋅2 C_7_H_8_, **5**, which was characterised by single crystal X‐ray crystallography, ^1^H NMR spectroscopy and TGA analyses. The framework transformation occurs when the intermediate MOF, DMOF‐1‐NHCH_2_OCH_3_, dissolves in the presence of pyrazole and re‐precipitates **5**. In contrast, the Mannich reaction of DMOF‐1‐NH_2_ with imidazole afforded a product, **6**, bearing the same gross structure as DMOF‐1‐NH_2_, showing that substituting pyrazole for imidazole prevents the dissolution of DMOF‐1‐NHCH_2_OCH_3_. This difference in reactivity has been rationalised on the basis of a functionality‐dependent dissolution process, in which the “free” nitrogen atom on pyrazole is in a position to compete with the dabco ligand for coordination to zinc, whereas the equivalent atom on imidazole is too far away to coordinate.

Subjecting MIL‐68(In)‐NH_2_ to a similar PSM reaction with pyrazole, gave a modified product **7** that did not contain the heterocycle. The first step of the Mannich reaction proceeded, but the methoxymethyl amine intermediate did not react with pyrazole in the expected manner. The X‐ray crystal structure of **7** suggests that this is a consequence of the location and orientation of these groups which are inaccessible to the pyrazole molecules, thus preventing the second step in the Mannich reaction from occurring.

This work has demonstrated that the post‐synthetic Mannich reaction represents a versatile route to introducing complex functionalities into a range of metal‐organic frameworks, and we are currently working to further develop the breadth of this approach.

## Experimental Section

Full experimental details are presented in the electronic supplementary information. As an example, the reaction of UiO‐66‐NH_2_ with formaldehyde and imidazole is presented here. UiO‐66‐NH_2_ (117 mg, 0.4 mmol eq. of NH_2_) and paraformaldehyde (24 mg, 0.8 mmol, 2 equiv.) were added into a glass vial containing methanol (5 mL). The vial was placed in an oven and heated at 50 °C for 24 h. The powder was then washed with methanol (three times) via centrifugation to remove any residual paraformaldehyde in the pores or on the solid surfaces. The powder was subsequently treated with imidazole (54 mg, 0.8 mmol, 2 equiv.) in 1,4‐dioxane at 80 °C for 24 h before quenching the reaction by rinsing the sample with fresh 1,4‐dioxane. The product was soaked in 1,4‐dioxane for 3 days, replacing the solvent with fresh solvent every 24 h, before isolation by centrifugation. Prior to characterisation, samples were left to dry in air for 2 h to obtain free‐flowing powders.

Full details of the X‐ray crystal structures of **5** and **7**⋅0.8 dioxane are given in the Supplementary Information. The structures have also been deposited with the Cambridge Structural Database (CCDC https://summary.ccdc.cam.ac.uk/structure-summary?doi=10.1002/chem.201801419 contains the supplementary crystallographic data for this paper. These data are provided free of charge by http://www.ccdc.cam.ac.uk/).

## Conflict of interest

The authors declare no conflict of interest.

## Supporting information

As a service to our authors and readers, this journal provides supporting information supplied by the authors. Such materials are peer reviewed and may be re‐organized for online delivery, but are not copy‐edited or typeset. Technical support issues arising from supporting information (other than missing files) should be addressed to the authors.

Supplementary
