# Supplementary material for: Post‐Synthetic Mannich Chemistry on Metal‐Organic Frameworks: System‐Specific Reactivity and Functionality‐Triggered Dissolution
Source: Chemistry. 2018 Jun 26;24(43):11094–102. doi: 10.1002/chem.201801419 (PMC6099314; doi:10.1002/chem.201801419)
Supplement: Supplementary file 1 — Supplementary [file CHEM-24-11094-s001.pdf]

CHEMISTRY
A European Journal

Supporting Information

Post-Synthetic Mannich Chemistry on Metal-Organic Frameworks: System-Specific Reactivity and Functionality-Triggered Dissolution

Harina Amer Hamzah,[a] William J. Gee,[a, b] Paul R. Raithby,[a] Simon J. Teat,[c]
Mary F. Mahon,*[a] and Andrew D. Burrows*[a]

chem_201801419_sm_miscellaneous_information.pdf

Post-synthetic Mannich chemistry on metal-organic frameworks: system-specific reactivity and functionality-triggered dissolution

Harina Amer Hamzah, William J. Gee, Paul R. Raithby, Simon J. Teat, Mary F. Mahon and Andrew D. Burrows

Supplementary information

1.	General experimental details	S2
2.	MOF structures	S3
3.	Syntheses of **1**-**3a**	S3
4.	Thermogravimetric analyses	S13
5.	Gas adsorption studies on **1**-**3** and **3a**	S13
6.	Mercury uptake studies on **3a**	S14
7.	Syntheses of **4**-**7**	S15
8.	Crystal structure determination	S30
9.	References	S35

1. General experimental details

All reagents and solvents were purchased from commercial sources and used without further purification.

Powder X-ray diffraction (PXRD) patterns for all samples were recorded on a Bruker AXS D8 Advance diffractometer with copper K_α radiation (λ = 1.5406 Å) at 298 K. The beam slit was set to 1 mm, detector slit set to 0.2 mm and anti-scattering slit set to 1 mm. Samples were dried in ambient conditions and ground using a pestle and mortar. The powders were packed onto a flat plate and measured with a 2θ range of 5 – 60°. The step size was 0.024° with the scan speed set to 0.3 s per step.

^{1}H NMR spectra were recorded on the digested MOFs at 298 K on a Bruker Avance 300 MHz Ultrashield NMR spectrometer. All ^{1}H NMR spectra were referenced to the residual *protio* peaks at δ 2.50 ppm for DMSO-d_6 with the exception of MIL-68-(In) materials, which were referenced to the residual *protio* peaks at δ 4.80 ppm for D_2O. Samples were dried at 100 °C for 15 minutes prior to digestion. For the UiO-66 materials, a typical MOF digestion was carried out by adding 10 mg of a crystalline sample into 0.4 mL of DMSO-d_6 and 0.2 mL of a stock solution of NH_4F in D_2O (4.14 M). For the IRMOF and DMOF materials, the MOF digestions were each carried out using approximately 10 mg of crystalline sample in 0.4 mL of DMSO-d_6 and 0.2 mL of a stock solution of 0.1 mL of 35 wt% DCl/D_2O in 3 mL DMSO. For the MIL-68(In) materials, the MOF digestion was carried out with approximately 10 mg of crystalline sample in 0.4 mL of D_2O and 0.2 mL of a stock solution of 0.1 mL of 30 wt% NaOD/D_2O in 3 mL D_2O. All cases, the mixtures were sonicated until all solids had completely dissolved. COSY spectra were used to fully assign the signals for the digested products.

Mass spectra was recorded on digested MOF solutions diluted in EtOH, using a Bruker micrOTOF electrospray ionisation time-of-flight (ESI-TOF) mass spectrometer. Atomic emission spectroscopy was carried out by Mr Alan Carver on a Perkin Elmer 3100 instrument. FTIR spectra were recorded on solid samples using a PerkinElmer Spectrum 100 spectrometer mounted on a diamond/gem platform.

TGA was carried out on the solid samples using a Setaram Setsys Evolution 16/18 thermogravimetric analyser. The samples were heated from 30 °C to 600 °C at a rate of 20 K/min under a flow of argon gas (20 mL/min).

A typical gas sorption measurement was carried out by loading an approximately 20 mg of MeOH rinsed sample into a pre-weighed sample tube. The sample was then heated at 120 °C for 12 hours on a BELSORP Mini-II (BEL Japan) gas sorption analyser. The sample tube was re-weighed to obtain a consistent mass of the activated sample prior to the gas adsorption measurement. To determine the specific surface area, N_2 sorption isotherm was recorded at 77 K. The temperature was kept constant using a liquid nitrogen bath. The specific surface area was calculated based on the Brunauer-Emmett-Teller (BET) method in the p/p_0 range of 0.05 - 0.10. CO_2 and N_2 adsorption isotherms were also recorded at 273 K. The selectivity of CO_2 over N_2 of the material was calculated from the single gas isotherms by dividing the CO_2 uptake by that of N_2 at a specific partial pressure (0.1 or 1.0 bar).

2. MOF structures

The structures of the parent frameworks for the MOFs used in this study – $[Zr_6O_4(OH)_4(bdc)_6]$ (UiO-66),[S1] $[Zn_4O(bdc)_3]$ (IRMOF-1/MOF-5),[S2] $[Zn_2(bdc)_2(dabco)]$ (DMOF-1)[S3] and [In(OH)(bdc)] (MIL-68(In))[S4] – are depicted in Figure S1.

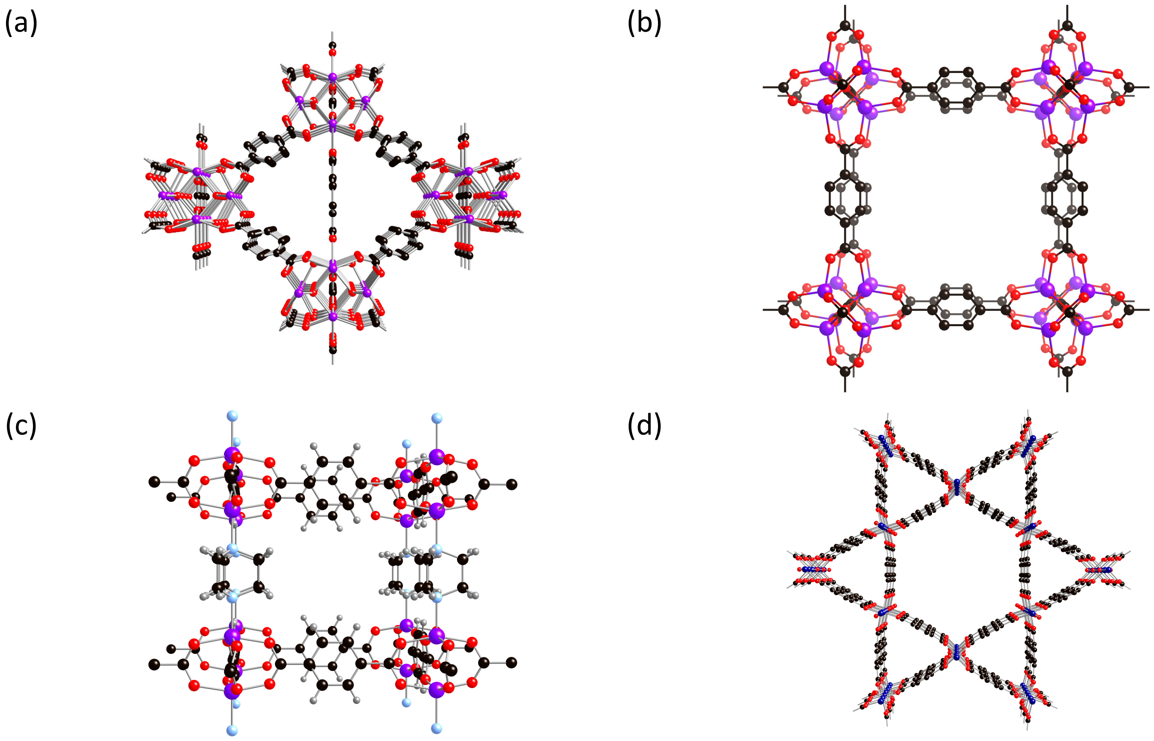

Figure S1. The structures of (a) UiO-66, (b) IRMOF-1, (c) DMOF-1 and (d) MIL_68(In).

3. Syntheses of 1-3a

3.1 Synthesis of UiO-66-NH_2

The synthesis was modified from a procedure reported by Garibay and Cohen.[S5] In a typical reaction, H_2bdc-NH_2 (190 mg, 1.05 mmol) along with $ZrCl_4$ (243 mg, 1.05 mmol) and DMF (12 mL) were loaded into a Teflon-lined autoclave. The solution was stirred until the reactants had completely dissolved. The autoclave was placed in an oven and heated at 120 °C for 24 h. The resulting yellow powder was rinsed and centrifuged with MeOH (6000 rpm for 15 min) to remove unreacted H_2bdc-NH_2 and residual DMF in the pores. The washing procedure was repeated over 3 days with the solvent replaced every 24 h. Finally, the UiO-66-NH_2 powder was dried under vacuum at 120 °C for 12 h. Prior to post-synthetic modification reactions, UiO-66-NH_2 was washed with 1,4-dioxane by centrifugation over 3 days with the solvent replaced every 24 h. The powder was then left to dry under ambient conditions. ^{1}H NMR (NH_4F/D_2O/DMSO-d_6): 7.56d (1H), 7.12s (1H), 7.05d (1H).

3.2 Synthesis of UiO-66-$NHCH_2$pyz, 1

UiO-66-NH_2 (117 mg, ca. 0.4 mmol eq. of NH_2) and paraformaldehyde (24 mg, 0.8 mmol, 2 eq.) were added into a glass vial containing methanol (5 mL). The vial was placed in an oven and heated at 50 °C for 24 h. The powder was then washed with methanol (three times) *via* centrifugation to remove any residual paraformaldehyde in the pores or on the solid surfaces. The powder was subsequently treated with pyrazole (54 mg, 0.8 mmol, 2 eq.) in 1,4-dioxane at 80°C for 24 h before quenching the reaction by rinsing the sample with fresh 1,4-dioxane. The product was soaked in 1,4-dioxane for 3 days, replacing the solvent with fresh solvent every 24 h, before isolation by centrifugation. Prior to characterisation, samples were left to dry in air for 2 h to obtain free-flowing powders. The PXRD pattern for **1** is shown in Figure S2, the ^{1}H NMR spectrum of digested **1** is shown in Figure S3, the ESI mass spectrum of digested **1** is shown in Figure S4 and the FTIR spectrum of **1** is shown in Figure S5.

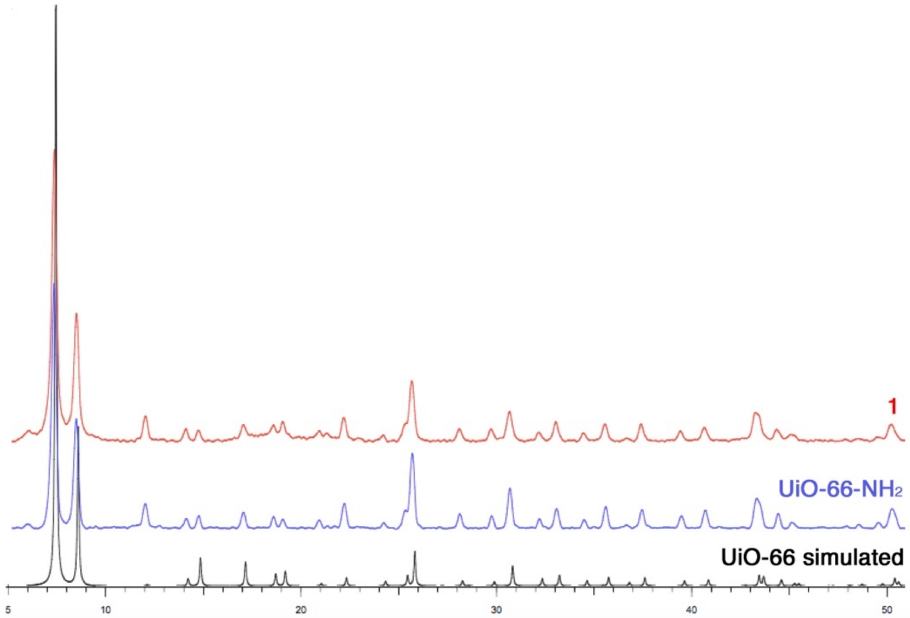

Figure S2. The PXRD pattern for **1** in comparison with experimental PXRD pattern for UiO-66-NH_2 and the simulated pattern for UiO-66.

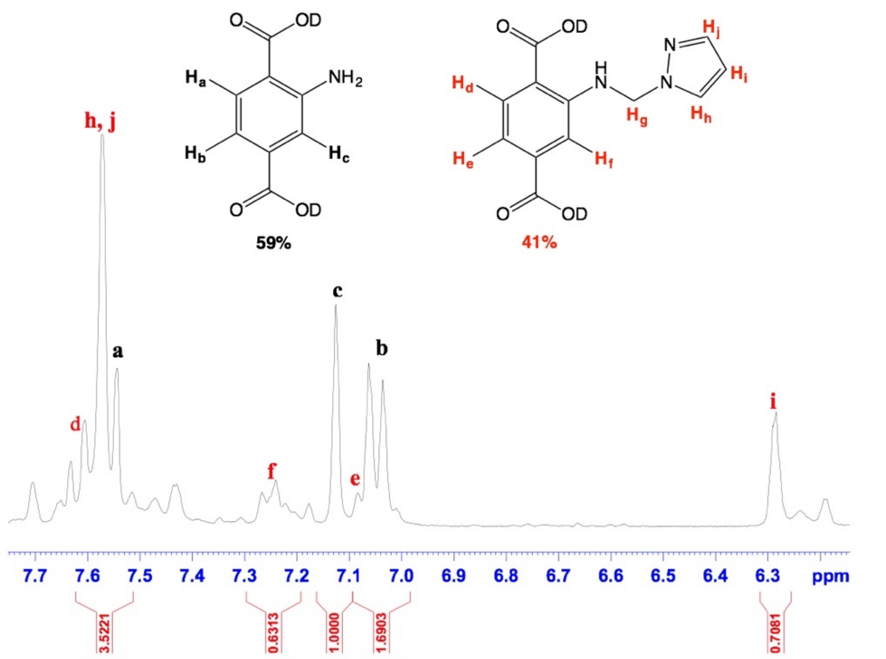

Figure S3. The ^{1}H NMR spectrum of **1** following digestion in NH_4F/D_2O and DMSO-d_6.

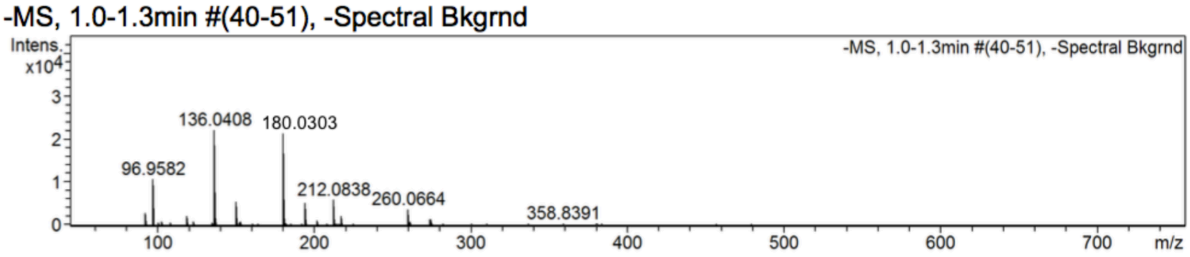

#	m/z	I	I %	Area	S/N
1	92.0479	2831	12.9	59	519.6
2	96.9582	10647	48.4	223	1871.0
3	136.0408	21980	100.0	603	3906.1
4	150.0562	5366	24.4	151	1087.3
5	180.0303	21230	96.6	719	4834.9
6	181.0345	2234	10.2	65	508.1
7	194.0457	5095	23.2	166	1139.1
8	212.0838	5864	26.7	77	1304.5
9	216.9084	2221	10.1	88	519.0
10	260.0664	3682	16.8	159	1535.2

Figure S4. The negative ion ESI mass spectrum of **1** following digestion in NH_4F/H_2O.

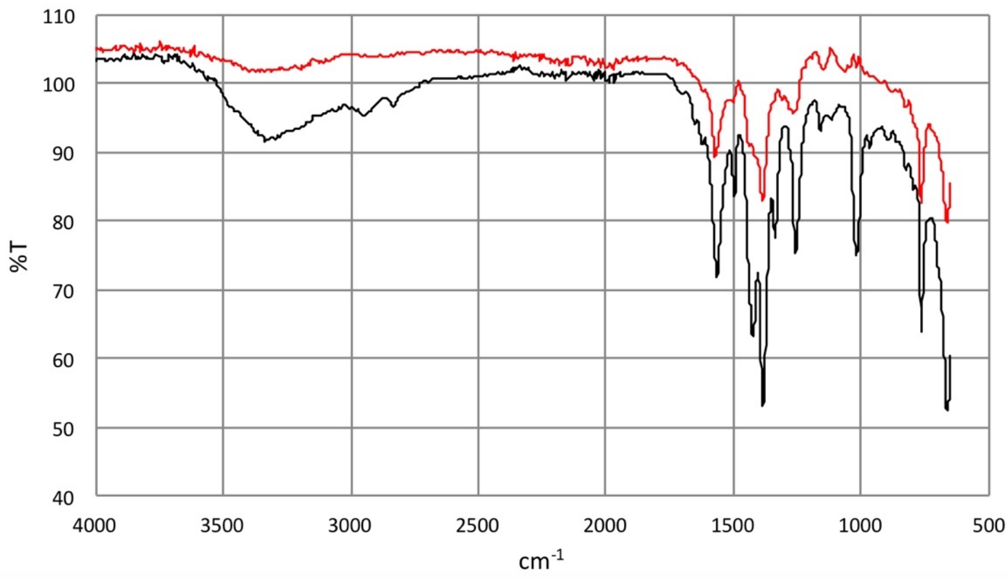

Figure S5. The FT-IR spectrum of **1** (red) in comparison to that of UiO-66-NH_2(black).

3.3 Synthesis of UiO-66-NHCH$_2$im, 2

UiO-66-NH$_2$ (117 mg, ca. 0.4 mmol eq. of NH$_2$) and paraformaldehyde (24 mg, 0.8 mmol, 2 eq.) were added into a glass vial containing methanol (5 mL). The vial was placed in an oven and heated at 50 °C for 24 h. The powder was then washed with methanol (three times) *via* centrifugation to remove any residual paraformaldehyde in the pores or on the solid surfaces. The powder was subsequently treated with imidazole (54 mg, 0.8 mmol, 2 eq.) in 1,4-dioxane at 80°C for 24 h before quenching the reaction by rinsing the sample with fresh 1,4-dioxane. The product was soaked in 1,4-dioxane for 3 days, replacing the solvent with fresh solvent every 24 h, before isolation by centrifugation. Prior to characterisation, samples were left to dry in air for 2 h to obtain free-flowing powders. The PXRD pattern for **2** is shown in Figure S6, the ^{1}H NMR spectrum of digested **2** is shown in Figure 1, the ESI mass spectrum of digested **2** is shown in Figure S7 and the FTIR spectrum of **2** is shown in Figure S8.

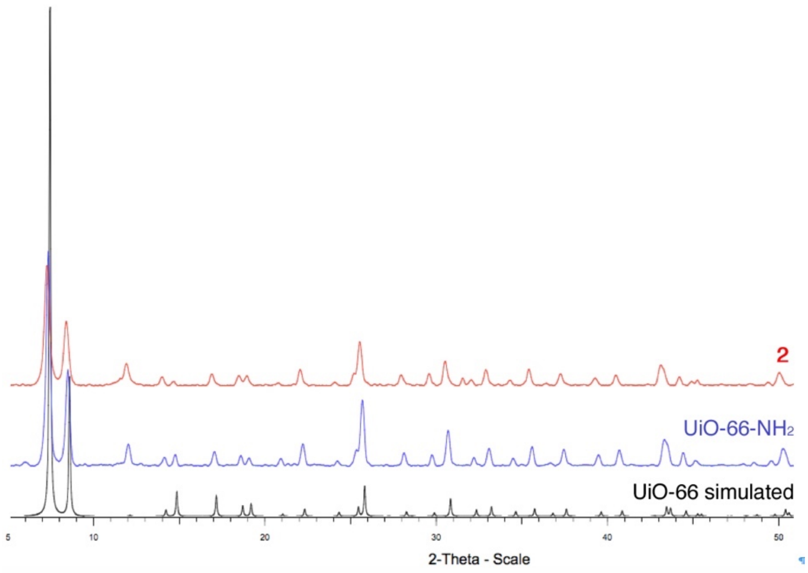

Figure S6. The PXRD pattern for **2** in comparison with experimental PXRD pattern for UiO-66-NH$_2$ and the simulated pattern for UiO-66.

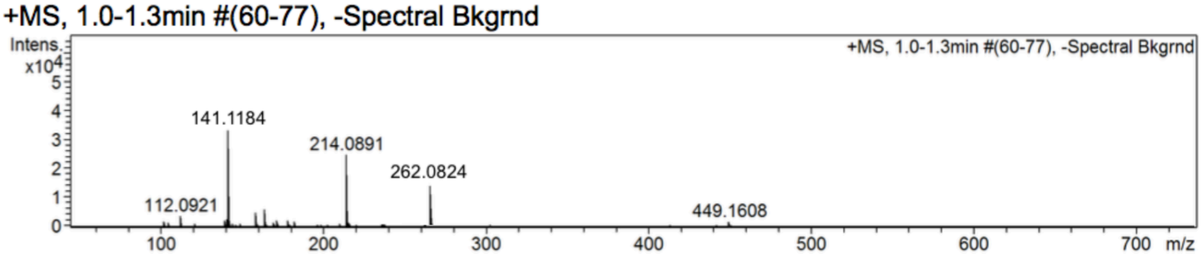

#	m/z	I	I %	Area	S/N
1	112.0921	3569	10.8	89	4688.1
2	139.0469	2048	6.2	58	524.7
3	141.0004	2399	7.3	36	578.8
4	141.1184	32988	100.0	818	7930.0
5	142.1199	2493	7.6	80	581.9
6	158.0470	4821	14.6	61	770.6
7	164.0364	5685	17.2	226	881.7
8	178.0516	1947	5.9	72	308.8
9	214.0891	24765	75.1	624	4429.2
10	262.0824	16242	49.2	462	2531.0

Figure S7. The positive ion ESI mass spectrum of **2** following digestion in NH_4F/H_2O.

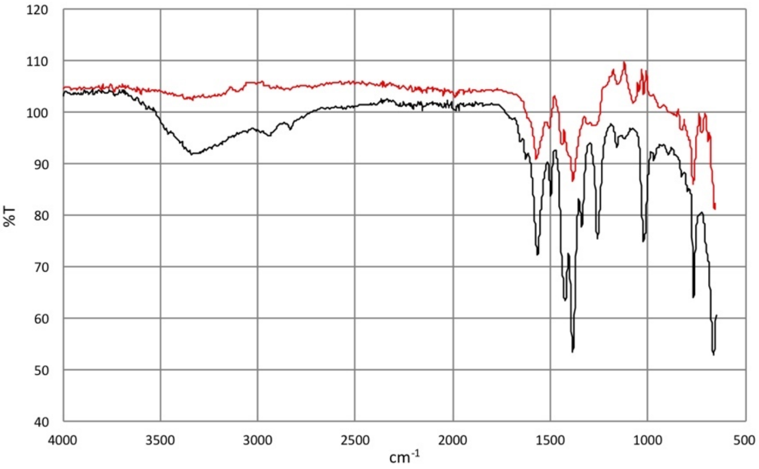

Figure S8. The FT-IR spectrum of **2** (red) in comparison to that of UiO-66-NH_2(black).

3.4 Synthesis of UiO-66-NHCH$_2$im-SH, 3

UiO-66-NH$_2$ (117 mg, ca. 0.4 mmol eq. of NH$_2$) and paraformaldehyde (24 mg, 0.8 mmol, 2 eq.) were added into a glass vial containing methanol (5 mL). The vial was placed in an oven and heated at 50 °C for 24 h. The powder was then washed with methanol (three times) *via* centrifugation to remove any residual paraformaldehyde in the pores or on the solid surfaces. The powder was subsequently treated with 2-mercaptoimidazole (80 mg, 0.8 mmol, 2 eq.) in 1,4-dioxane at 80°C for 24 h before quenching the reaction by rinsing the sample with fresh 1,4-dioxane. The product was soaked in 1,4-dioxane for 3 days, replacing the solvent with fresh solvent every 24 h, before isolation by centrifugation. Prior to characterisation, samples were left to dry in air for 2 h to obtain free-flowing powders. The PXRD pattern for **3** is shown in Figure S9, the ^{1}H NMR spectrum of digested **2** is shown in Figure S10, the ESI mass spectrum of digested **3** is shown in Figure S11 and the FTIR spectrum of **2** is shown in Figure S12.

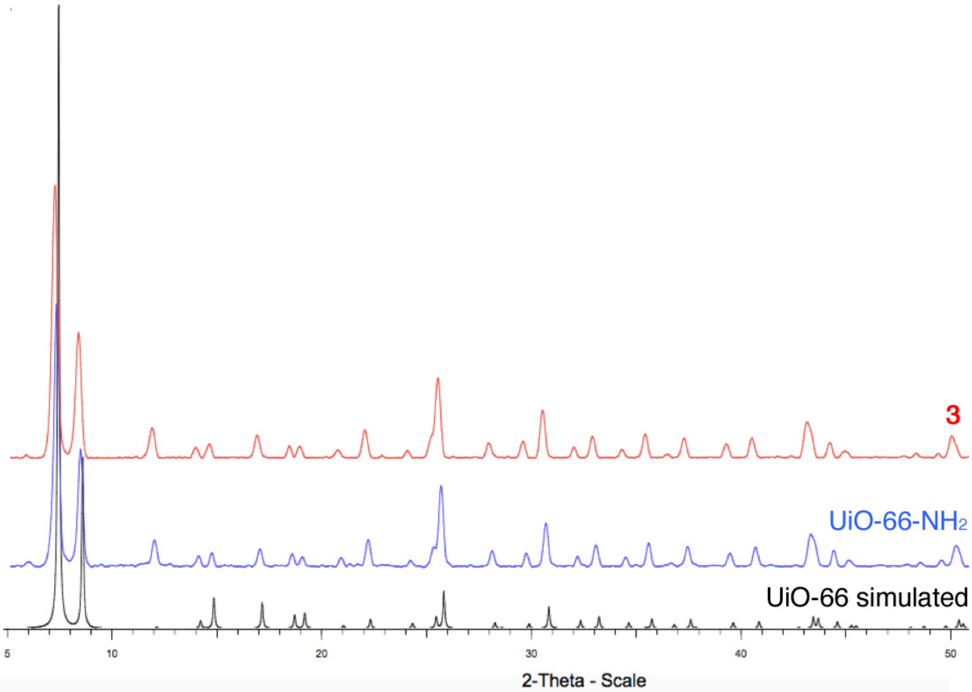

Figure S9. The PXRD pattern for **3** in comparison with experimental PXRD pattern for UiO-66-NH$_2$ and the simulated pattern for UiO-66.

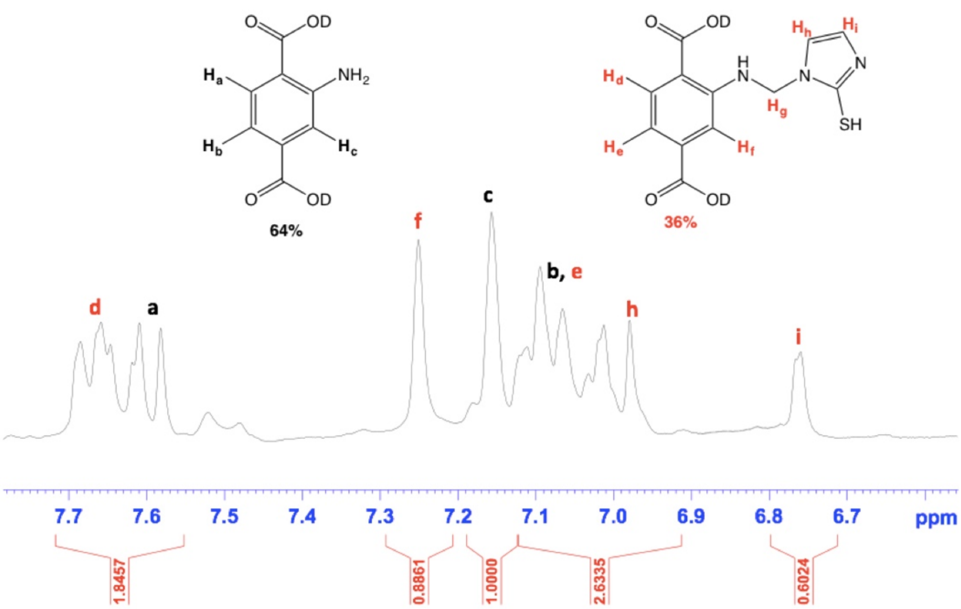

Figure S10. The ^{1}H NMR spectrum of **3** following digestion in NH_4F/D_2O and DMSO-d_6.

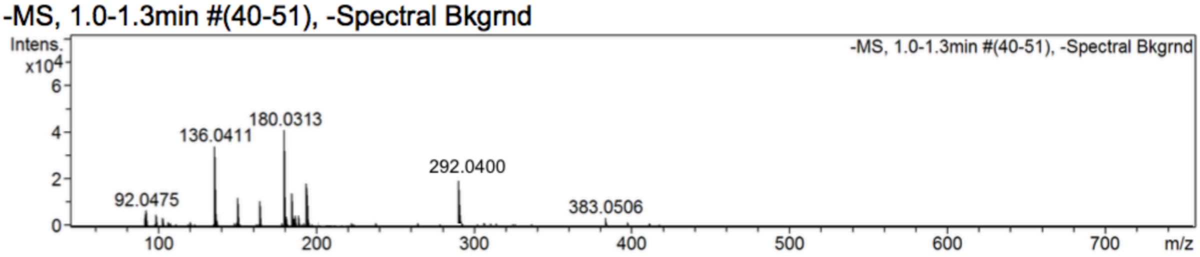

#	m/z	I	I %	Area	S/N
1	92.0475	6617	16.2	147	577.1
2	99.0001	5079	12.5	110	417.0
3	136.0411	34119	83.7	918	2448.6
4	150.0567	12073	29.6	343	861.9
5	164.0690	10261	25.2	419	731.4
6	180.0313	40753	100.0	1233	3040.2
7	184.8979	13664	33.5	456	1033.6
8	188.8991	4619	11.3	154	353.3
9	194.0463	18035	44.3	642	1401.9
10	292.0400	22342	54.8	723	1723.1

Figure S11. The negative ion ESI mass spectrum of **3** following digestion in NH_4F/H_2O.

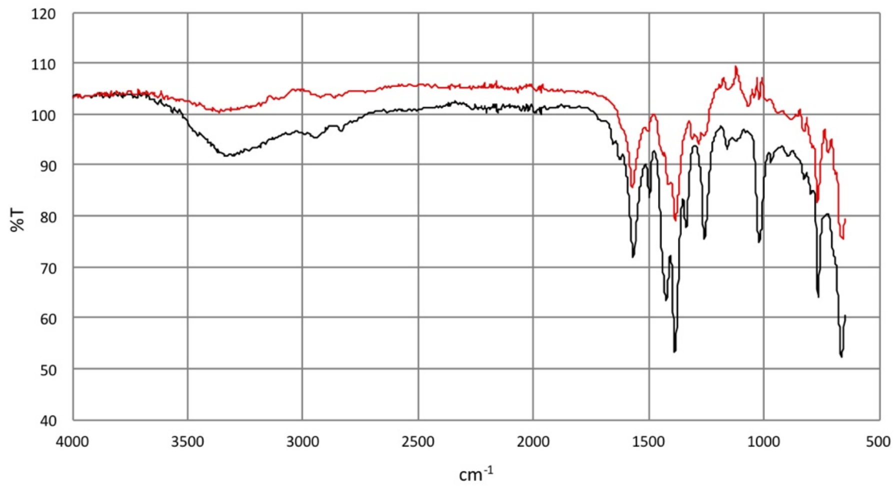

Figure S12. The FT-IR spectrum of **3** (red) in comparison to that of UiO-66-NH_2(black).

3.5 Synthesis of UiO-66-NHCH$_2$im-SH, 3a

UiO-66-NH$_2$ (117 mg, ca. 0.4 mmol eq. of NH$_2$) and paraformaldehyde (24 mg, 0.8 mmol, 2 eq.) were added into a glass vial containing methanol (5 mL). The vial was placed in an oven and heated at 50 °C for 24 h. The powder was then washed with methanol (three times) *via* centrifugation to remove any residual paraformaldehyde in the pores or on the solid surfaces. The powder was subsequently treated with 2-mercaptoimidazole (80 mg, 0.8 mmol, 2 eq.) in 1,4-dioxane at 50°C for 24 h before quenching the reaction by rinsing the sample with fresh 1,4-dioxane. The product was soaked in 1,4-dioxane for 3 days, replacing the solvent with fresh solvent every 24 h, before isolation by centrifugation. Prior to characterisation, samples were left to dry in air for 2 h to obtain free-flowing powders. The ^{1}H NMR spectrum of digested **3a** is shown in Figure S13.

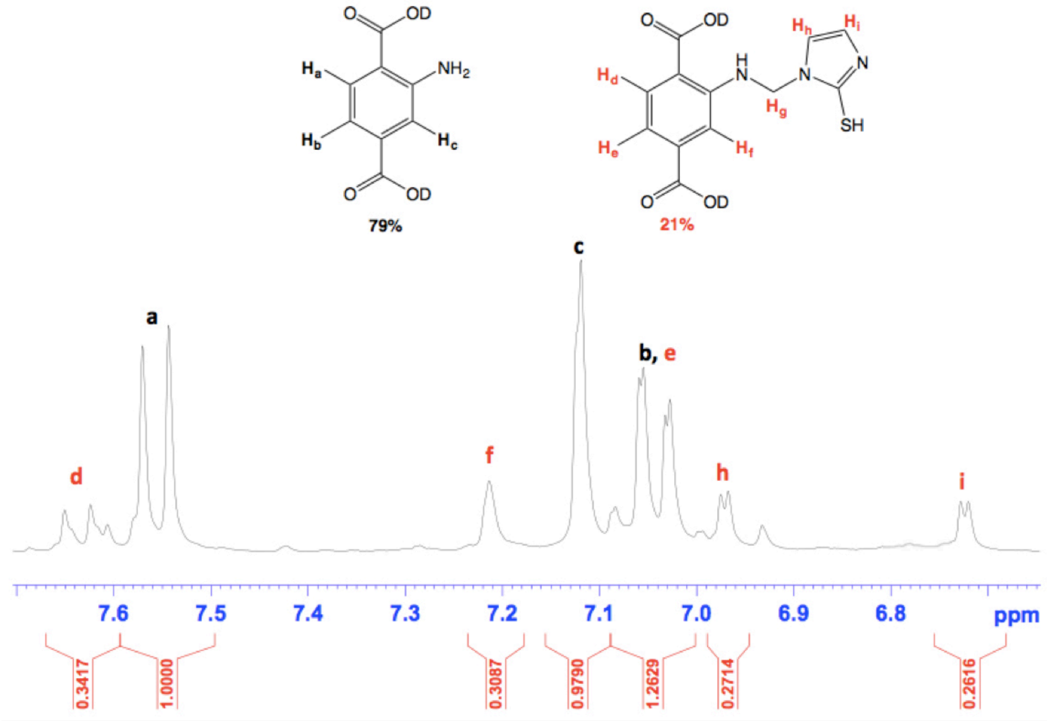

Figure S13. The ^{1}H NMR spectrum of **3a** following digestion in NH_4F/D_2O and DMSO-d_6.

4. Thermogravimetric analyses

Thermogravimetric analyses for UiO-66-NH_2 and compounds **1-3** and **3a** are shown in Figure S14.

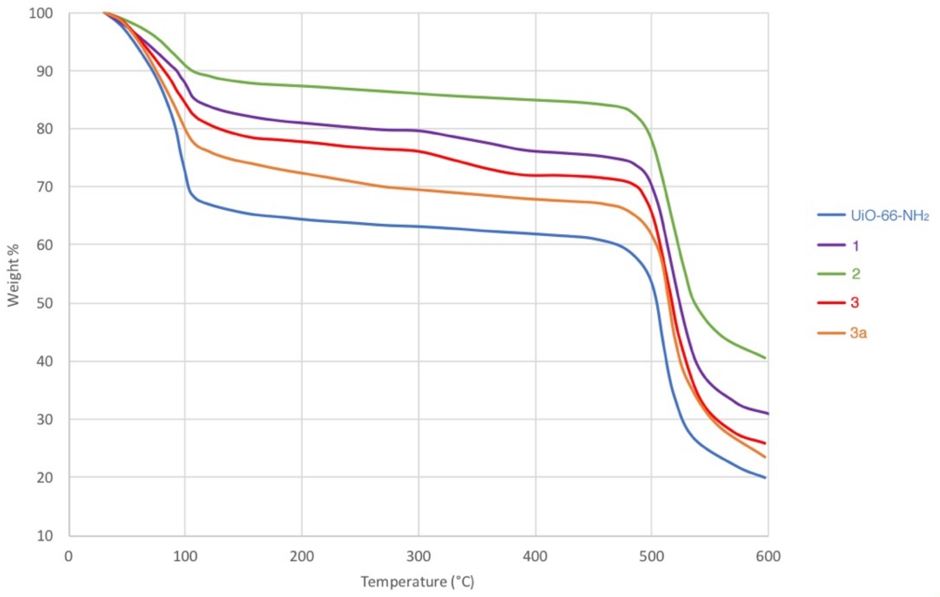

Figure S14. Thermogravimetric analyses for UiO-66-NH_2 and compounds **1-3** and **3a**.

5. Gas adsorption studies on 1-3 and 3a

Carbon dioxide and nitrogen sorption data at 273 K for UiO-66-NH_2 and compounds **1-3** and **3a** are shown in Figures S15 and S16 respectively.

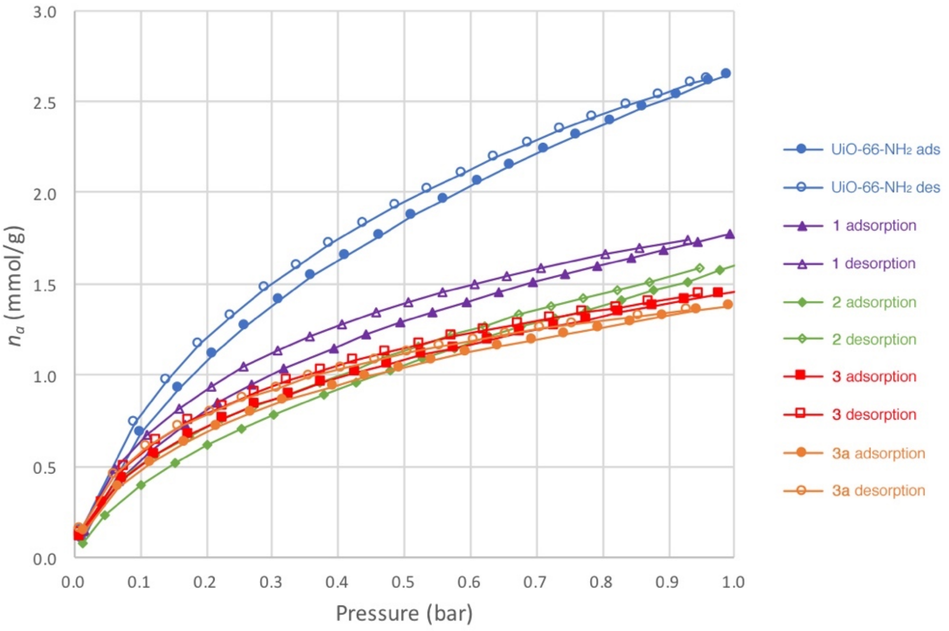

Figure S15. Carbon dioxide adsorption and desorption data for UiO-66-NH_2 and compounds **1-3** and **3a** at 273 K.

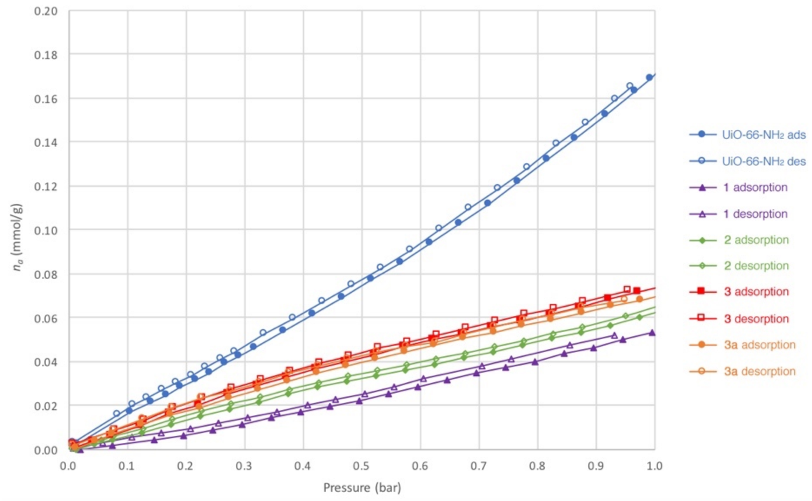

Figure S16. Nitrogen adsorption and desorption data for UiO-66-NH_2 and compounds **1-3** and **3a** at 273 K.

6. Mercury uptake studies on 3a

PXRD diffraction patterns for **3a** prior to and following treatment with mercury(II) chloride are shown in Figure S17.

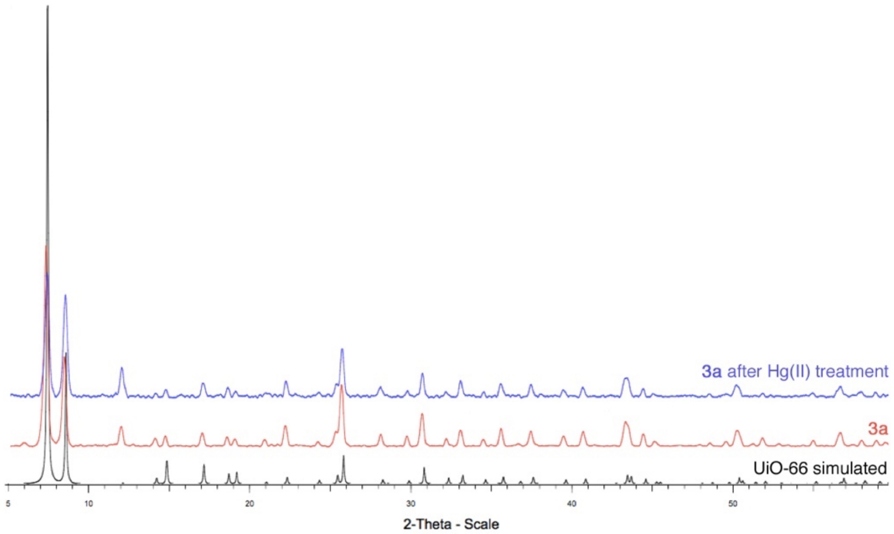

Figure S17. The PXRD pattern for **3a** prior to and following treatment with mercury(II) chloride in comparison with the simulated pattern for UiO-66.

7. Syntheses of 4-7

7.1 Synthesis of IRMOF-$NHCH_2$pyz, 4

IRMOF-3 was synthesised according to a previously reported procedure [^{1}H NMR (DCl/D_2O/DMSO-d_6): 7.79d (1H), 7.42d (1H), 7.08dd (1H)].[S6] In a typical PSM procedure, IRMOF-3 crystals (108 mg, ca. 0.4 mmol eq. of NH_2), paraformaldehyde (24 mg, 0.8 mmol, 2 eq.) and MeOH (32 µL, 0.8 mmol, 2 eq.) were added into a glass vial containing 5 mL toluene. The vial was sealed, placed in an oven and heated at 50 °C for 24 h. The crystals were then washed with fresh toluene (three times) to remove any residual paraformaldehyde and MeOH in the pores or on the solid surfaces. The crystals were subsequently treated with pyrazole (54 mg, 0.8 mmol, 2 eq.) in toluene at 80 °C for 24 h before quenching the reaction by rinsing the crystals with fresh toluene. The crystals were soaked in toluene for 3 days, replacing the solvent with fresh solvent every 24 h. The crystals were then isolated *via* filtration. The crystals were stored under an inert atmosphere to avoid degradation. The PXRD pattern for **4** is shown in Figure S18, the ^{1}H NMR spectrum of digested **4** is shown in Figure S19, the ESI mass spectrum for digested **4** is shown in Figure S20 and the FTIR spectrum of **4** is shown in Figure S21.

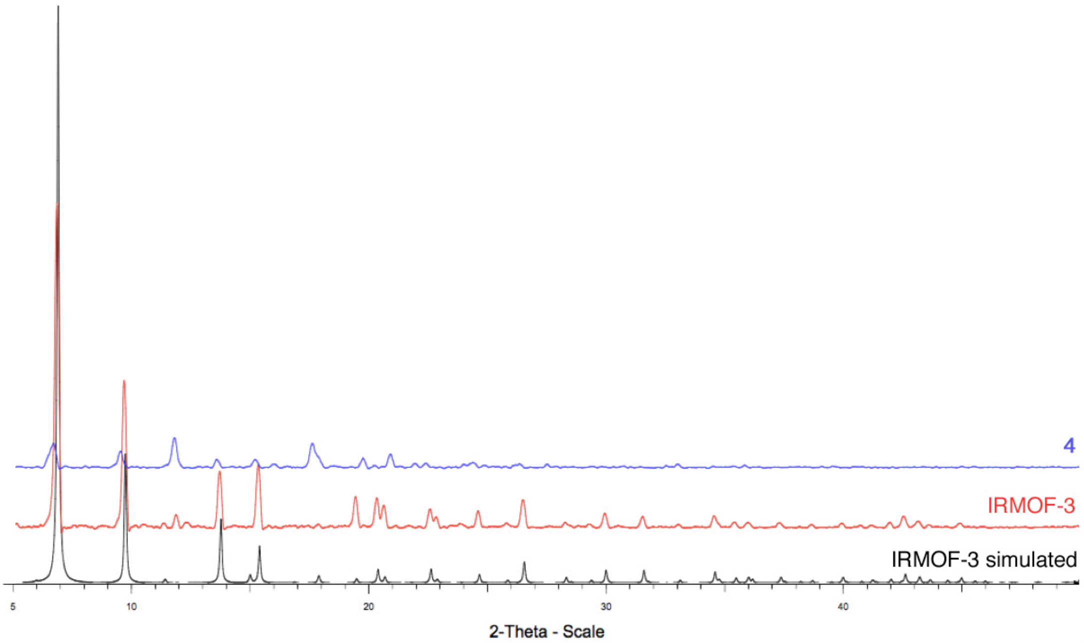

Figure S18. The PXRD pattern for **4** in comparison with experimental and simulated PXRD patterns for IRMOF-3.

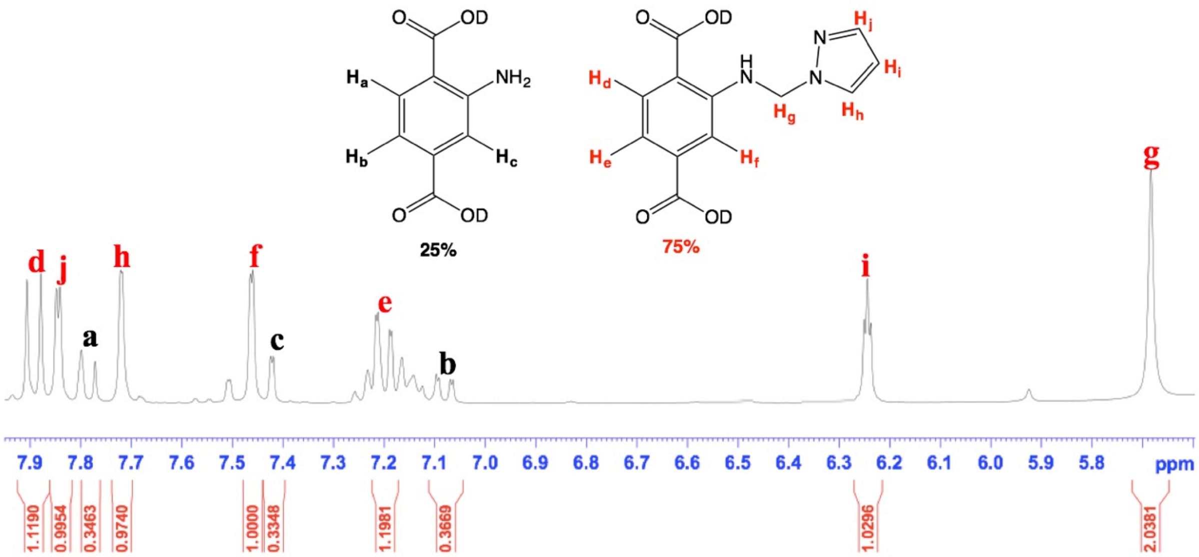

Figure S19. The ^{1}H NMR spectrum of **4** following digestion in DCl/D_2O and DMSO-d_6.

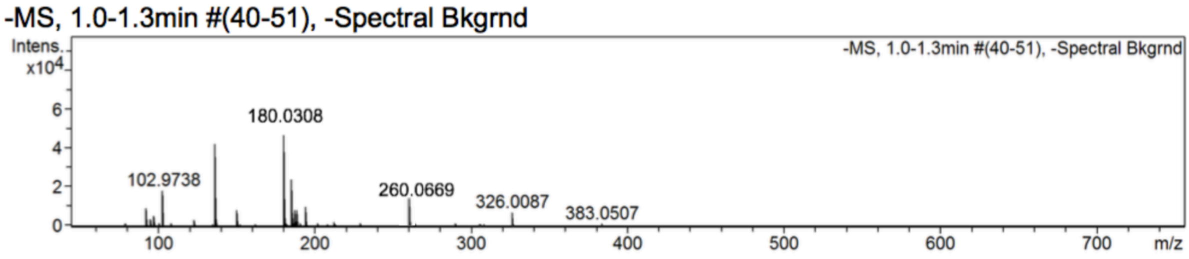

#	m/z	I	I %	Area	S/N
1	92.0470	9004	18.5	200	936.8
2	102.9738	18249	37.6	425	1729.9
3	136.0411	42299	87.1	1187	3915.0
4	150.0567	8295	17.1	247	811.2
5	180.0308	48575	100.0	1601	6096.3
6	184.8976	23665	48.7	821	3165.8
7	186.8978	7864	16.2	254	1081.2
8	188.8994	7865	16.2	275	1112.4
9	260.0669	16289	33.5	314	1463.7
10	326.0087	7061	14.5	364	2576.0

Figure S20. The negative ion ESI mass spectrum of **4** following digestion in NH_4F/H_2O.

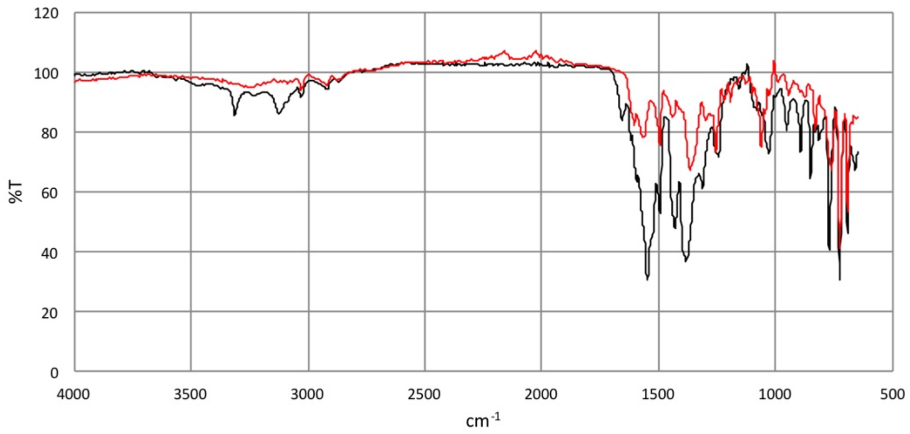

Figure S21. The FT-IR spectrum of **4** (red) in comparison to that of IRMOF-3 (black).

7.2 Synthesis of [$Zn_3(bdc\text{-}NH_2)_{1.32}(bdc\text{-}NHCH_2pyz)_{1.68}(dabco)$]·$2C_7H_8$, 5

DMOF-1-NH_2 was synthesised according to a previously reported procedure [^{1}H NMR ($DCl/D_2O/DMSO\text{-}d_6$): 7.82d (2H), 7.48d (2H), 7.13dd (2H), 3.60s (12H)].[S7] In a typical PSM procedure, DMOF-1-NH_2 crystals (120 mg, ca. 0.4 mmol eq. of NH_2), paraformaldehyde (24 mg, 0.8 mmol, 2 eq.) and MeOH (32 µL, 0.8 mmol, 2 eq.) were added into a glass vial containing 5 mL toluene. The vial was sealed, placed in an oven and heated at 50 °C for 24 h. The crystals were then washed with fresh toluene (three times) to remove any residual paraformaldehyde and MeOH in the pores or on the solid surfaces. The crystals were subsequently treated with pyrazole (54 mg, 0.8 mmol, 2 eq.) in toluene at 80 °C for 24 h before quenching the reaction by rinsing the crystals with fresh toluene. The crystals were soaked in toluene for 3 days, replacing the solvent with fresh solvent every 24 h. The crystals were then isolated *via* filtration. The PXRD pattern for **5** is shown in Figure S22 and the simulated PXRD pattern for **5** is shown in Figure S23. The ^{1}H NMR spectrum of digested **5** is shown in Figure S24, the ESI mass spectrum of digested **5** is shown in Figure S25 and the FTIR spectrum of **5** is shown in Figure S26. The PXRD patterns for DMOF-1-NH_2 treated with paraformaldehyde, methanol and pyrazole are shown in Figure S27, and the TGA for **5** is shown in Figure S28.

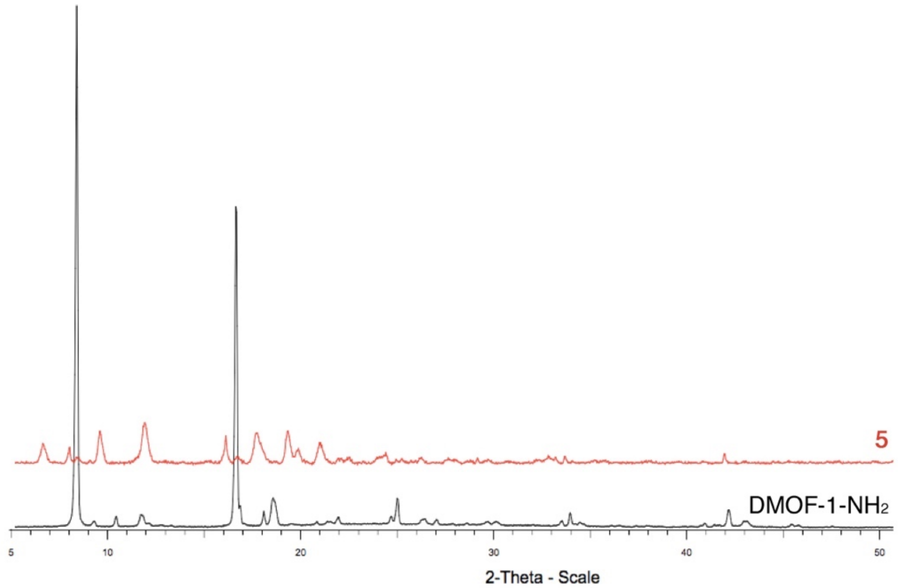

Figure S22. The PXRD pattern for **5** in comparison with that for DMOF-1-NH_2.

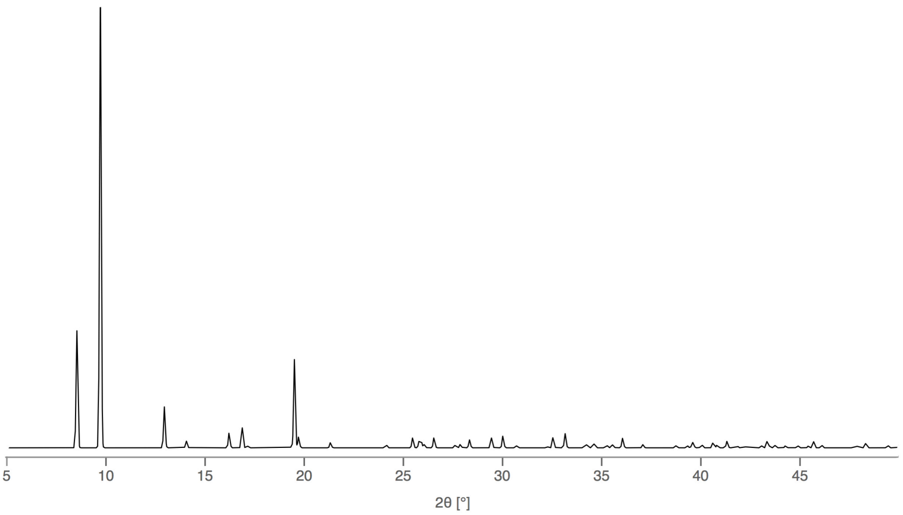

Figure S23. The simulated PXRD pattern derived from the crystal structure of **5**.

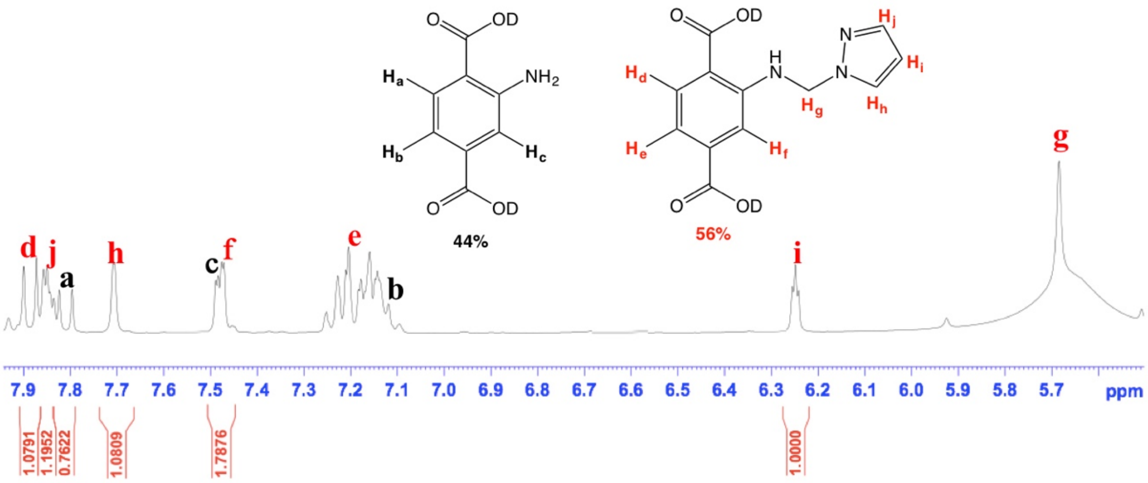

Figure S24. The ^{1}H NMR spectrum of **5** following digestion in DCl/D_2O and DMSO-d_6. A singlet for the 12 equivalent dabco protons is also observed at δ 3.60 ppm.

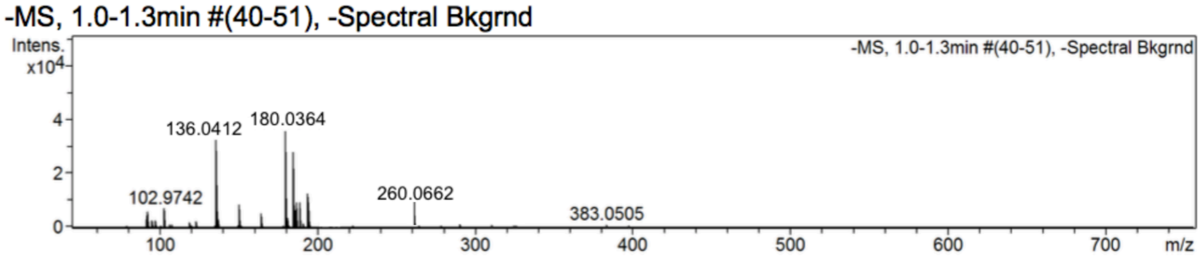

#	m/z	I	I %	Area	S/N
1	102.9742	7053	19.8	155	663.9
2	136.0412	32658	91.8	884	2743.9
3	150.0569	8253	23.2	222	679.3
4	180.0364	35573	100.0	1108	3342.5
5	184.8978	27950	78.6	968	2754.3
6	185.8986	6164	17.3	206	613.4
7	186.8980	9284	26.1	314	933.4
8	188.8991	9347	26.3	325	959.5
9	194.0461	12324	34.6	408	1336.9
10	260.0662	11503	32.3	454	1475.9

Figure S25. The negative ion ESI mass spectrum of **5** following digestion in NH_4F/H_2O.

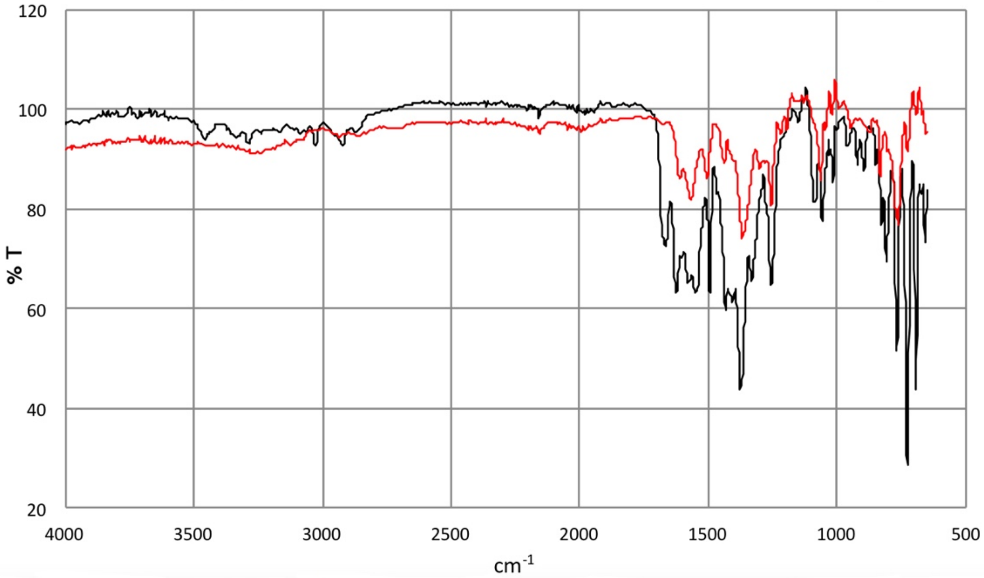

Figure S26. The FT-IR spectrum of **5** (red) in comparison to that of DMOF-1-NH_2 (black).

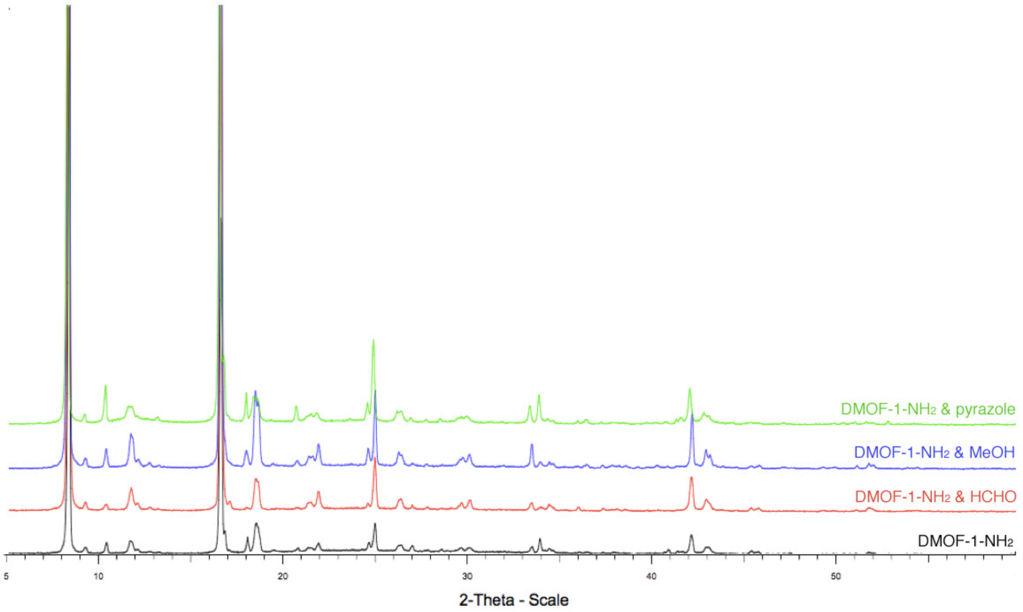

Figure S27. The PXRD pattern for **5** following treatment with paraformaldehyde, methanol and pyrazole.

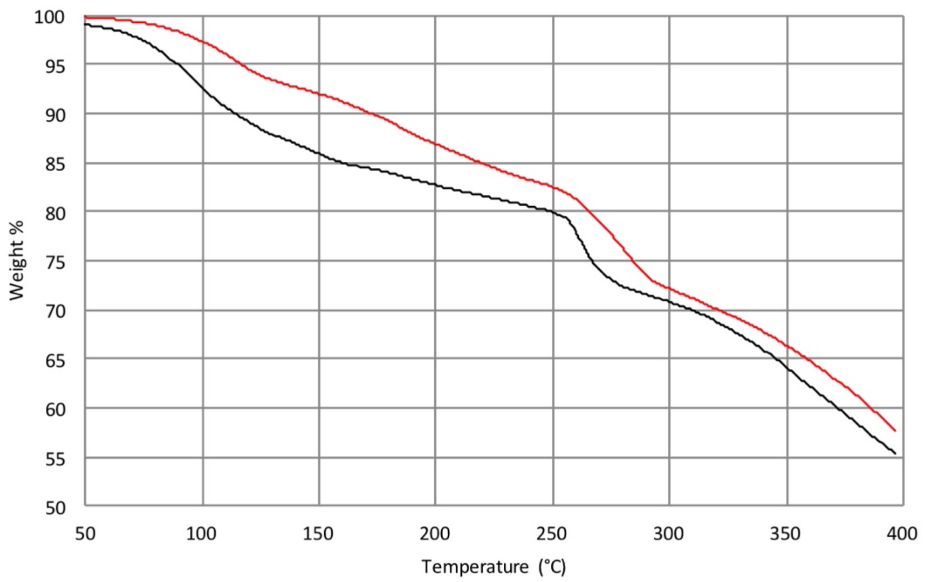

Figure S28. Thermogravimetric analyses for DMOF-1-NH_2 (black) and **5** (red).

7.3 Synthesis of DMOF-1-NHCH$_2$im, 6

In a typical PSM procedure, DMOF-1-NH$_2$ crystals (120 mg, ca. 0.4 mmol eq. of NH$_2$), paraformaldehyde (24 mg, 0.8 mmol, 2 eq.) and MeOH (32 µL, 0.8 mmol, 2 eq.) were added into a glass vial containing 5 mL toluene. The vial was sealed, placed in an oven and heated at 50 °C for 24 h. The crystals were then washed with fresh toluene (three times) to remove any residual paraformaldehyde and MeOH in the pores or on the solid surfaces. The crystals were subsequently treated with imidazole (54 mg, 0.8 mmol, 2 eq.) in toluene at 80 °C for 24 h before quenching the reaction by rinsing the crystals with fresh toluene. The crystals were soaked in toluene for 3 days, replacing the solvent with fresh solvent every 24 h. The crystals were then isolated *via* filtration. The PXRD pattern for **6** is shown in Figure S29, the ^{1}H NMR spectrum of digested **6** is shown in Figure S30, the ESI mass spectrum of **6** is shown in Figure S31 and the FTIR spectrum of **6** is shown in Figure S32. The TGA for **6** is shown in Figure S33.

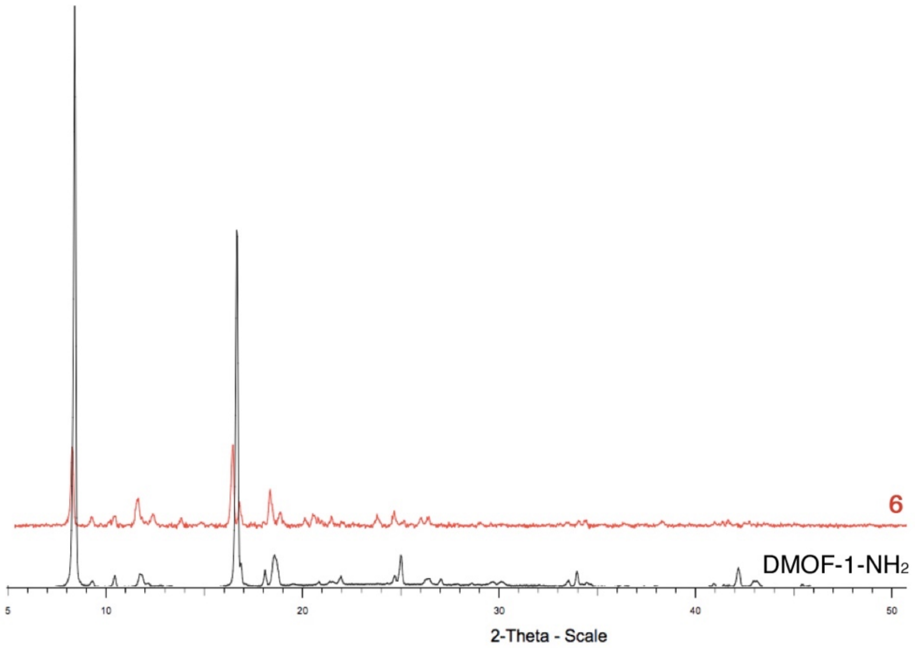

Figure S29. The PXRD pattern for **6** in comparison with that for DMOF-1-NH$_2$.

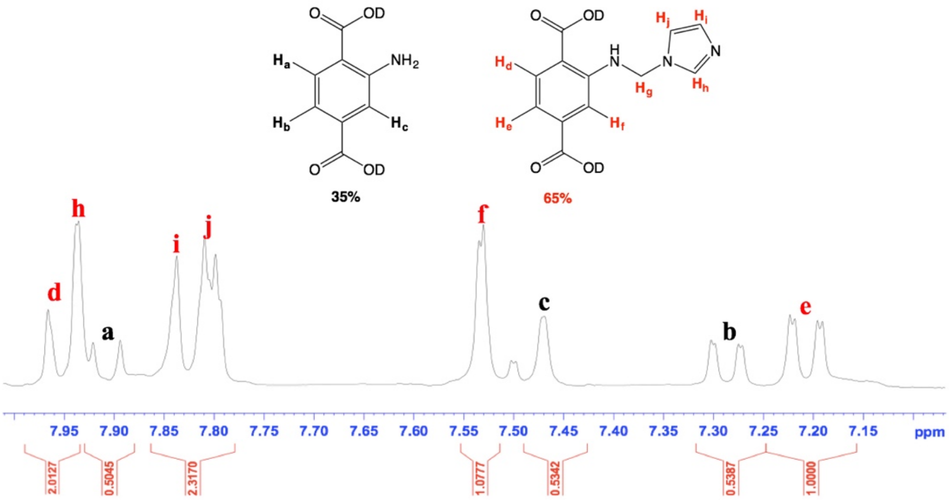

Figure S30. The ^{1}H NMR spectrum of **6** following digestion in DCl/D_2O and DMSO-d_6. A singlet for the 12 equivalent dabco protons is also observed at δ 3.60 ppm.

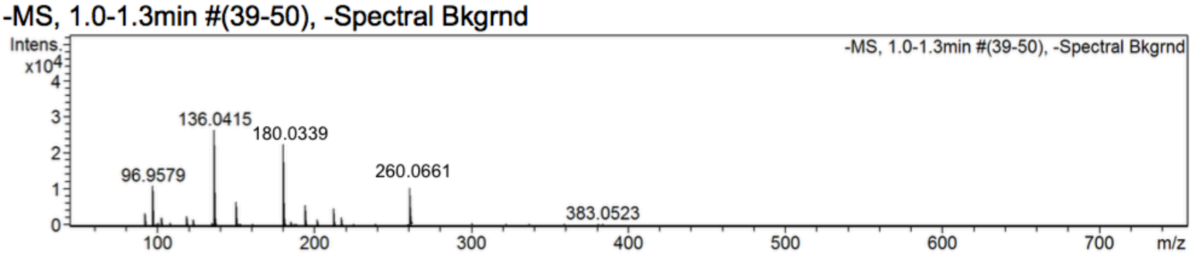

#	m/z	I	I %	Area	S/N
1	92.0471	3496	13.3	70	802.1
2	96.9579	10944	41.7	220	2378.6
3	118.9430	2592	9.9	57	479.9
4	136.0415	26253	100.0	672	5578.1
5	137.0462	2682	10.2	64	574.8
6	150.0569	6358	24.2	176	1536.5
7	180.0339	22481	85.6	727	6218.7
8	181.0355	2402	9.1	67	664.3
9	194.0465	5590	21.3	181	1544.4
10	260.0661	12273	46.7	250	2631.2

Figure S31. The negative ion ESI mass spectrum of **6** following digestion in NH_4F/H_2O.

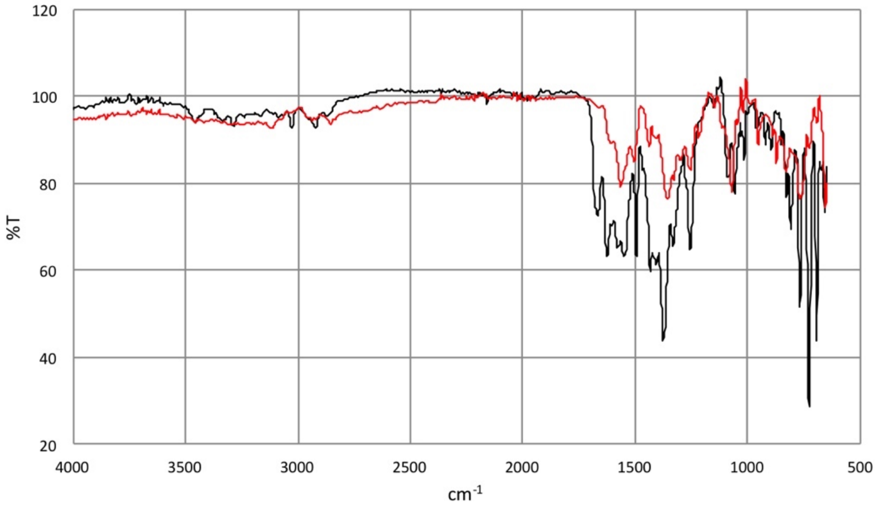

Figure S32. The FT-IR spectrum of **6** (red) in comparison to that of DMOF-1-NH_2 (black).

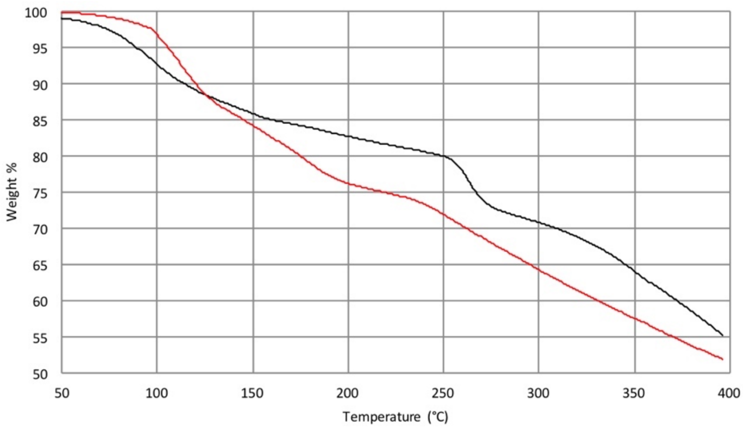

Figure S33. Thermogravimetric analyses for DMOF-1-NH_2 (black) and **6** (red).

7.4 Attempted synthesis of DMOF-1-$NHCH_2$imSH

In a typical PSM procedure, DMOF-1-NH_2 crystals (120 mg, ca. 0.4 mmol eq. of NH_2), paraformaldehyde (24 mg, 0.8 mmol, 2 eq.) and MeOH (32 µL, 0.8 mmol, 2 eq.) were added into a glass vial containing 5 mL toluene. The vial was sealed, placed in an oven and heated at 50 °C for 24 h. The crystals were then washed with fresh toluene (three times) to remove any residual paraformaldehyde and MeOH in the pores or on the solid surfaces. The crystals were subsequently treated with 2-mercaptoimidazole (80 mg, 0.8 mmol, 2 eq.) in toluene at 80 °C for 24 h before quenching the reaction by rinsing the crystals with fresh toluene. The crystals were soaked in toluene for 3 days, replacing the solvent with fresh solvent every 24 h. The crystals were then isolated *via* filtration. The PXRD pattern for the reaction product is shown in Figure S34 and the ^{1}H NMR spectrum of digested product is shown in Figure S35.

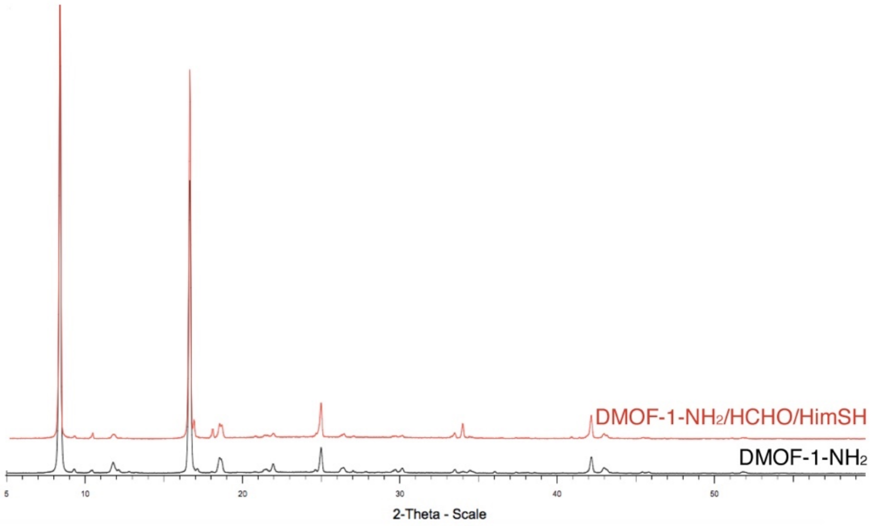

Figure S34. The PXRD pattern for the product from the reaction between DMOF-1-NH_2, formaldehyde and2-mercaptoimidazole in comparison with that for DMOF-1-NH_2.

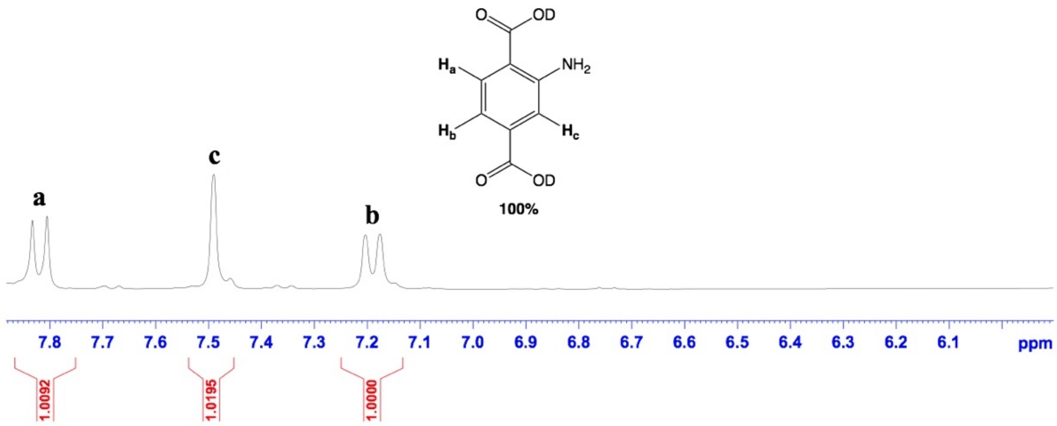

Figure S35. The ^{1}H NMR spectrum of for the product from the reaction between DMOF-1-NH_2, formaldehyde and 2-mercaptoimidazole following digestion in DCl/D_2O and DMSO-d_6.

7.5 Synthesis of MIL-68(In)-$NHCH_2OCH_3$, 7

MIL-68(In)-NH_2 was synthesised according to a previously reported procedure [^1H NMR ($NaOD/D_2O$): 7.65d (1H), 7.21s (1H), 7.13d (1H)].[S4] In a typical PSM procedure, crystals of MIL-68(In)-NH_2 crystals (124 mg, ca. 0.4 mmol eq. of NH_2) and paraformaldehyde (24 mg, 0.8 mmol, 2 eq.) were added into a glass vial containing 5 mL MeOH. The vial was sealed, placed in an oven and heated at 50 °C for 24 h. The crystals were then washed with 1,4-dioxane (three times) *via* filtration to remove any residual paraformaldehyde and MeOH in the pores or on the solid surfaces. The crystals were subsequently treated with pyrazole (54 mg, 0.8 mmol, 2 eq.) in 1,4-dioxane at 80 °C for 24 h before quenching the reaction by rinsing the sample with fresh 1,4-dioxane. The product was soaked in 1,4-dioxane for 3 days, replacing the solvent with fresh solvent every 24 h, before isolation by filtration. The PXRD pattern for **7** is shown in Figure S36, and the ^1H NMR spectrum of digested **7** is shown in Figure S37. The ^1H NMR spectrum of digested product from the reaction without pyrazole is shown in Figure S38, and the ESI mass spectrum for this material is shown in Figure S39. The FTIR spectrum of **7** is shown in Figure S40 and the TGA for **7** is shown in Figure S41.

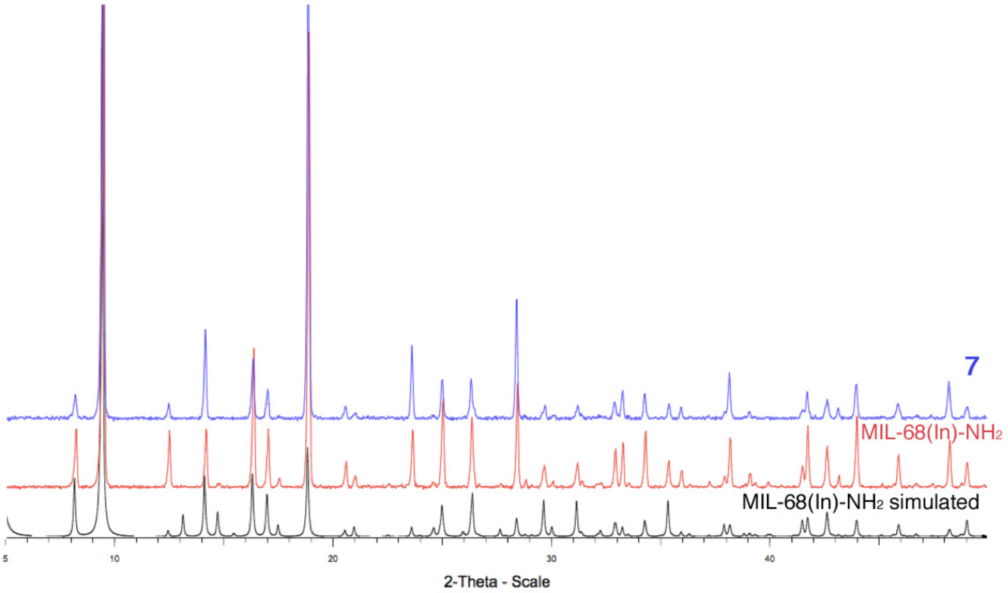

Figure S36. The PXRD pattern for **7** in comparison with that for MIL-68(In)-NH_2.

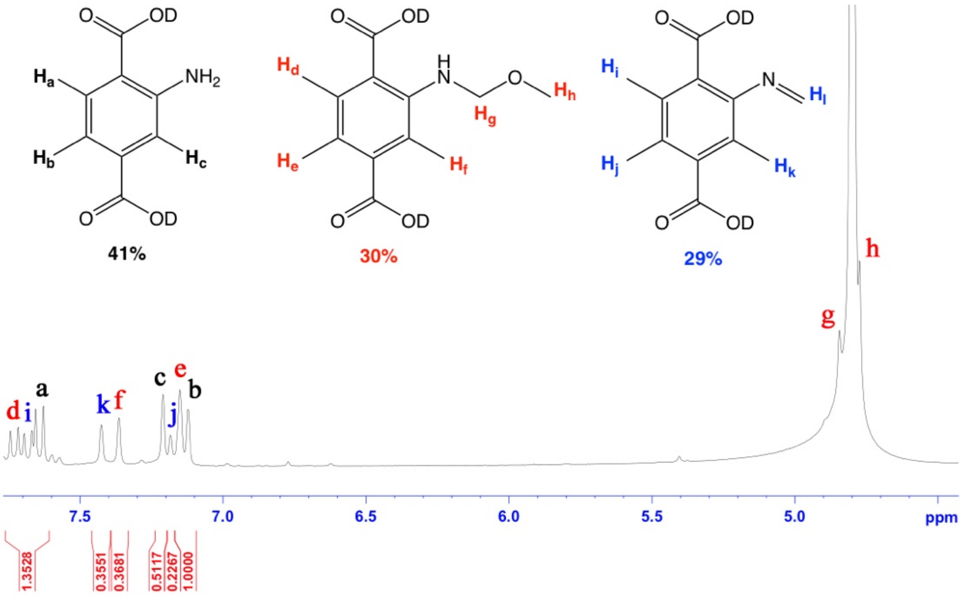

Figure S37. The ^{1}H NMR spectrum of **7** following digestion in $NaOD/D_2O$.

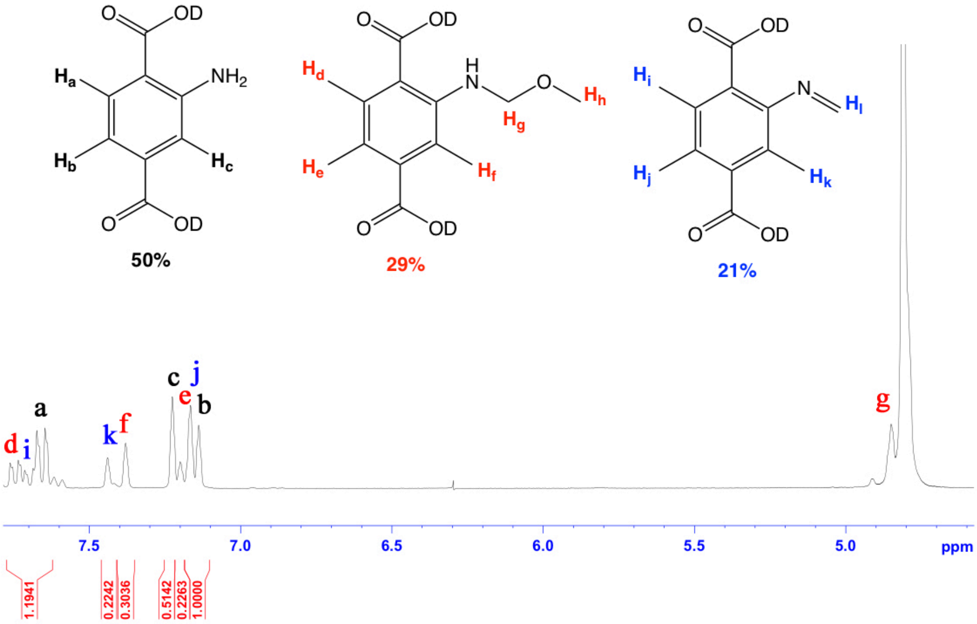

Figure S38. The ^{1}H NMR spectrum of the product of the reaction between MIL-68(In)-NH_2, formaldehyde and methanol, following digestion in $NaOD/D_2O$.

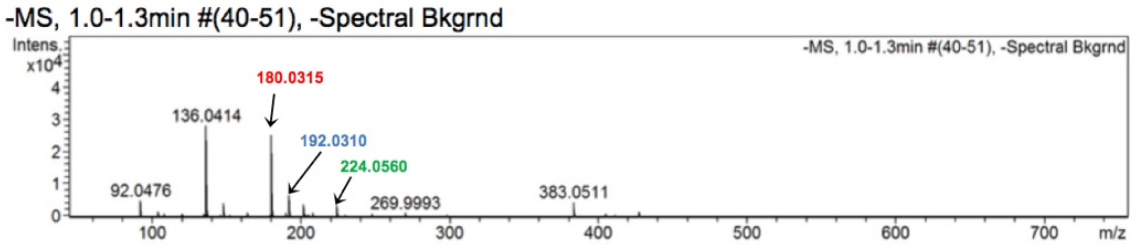

#	m/z	I	I %	Area	S/N
1	92.0476	4773	17.1	106	691.3
2	136.0414	27868	100.0	791	4233.0
3	137.0453	2390	8.6	61	365.5
4	148.0413	4122	14.8	117	695.9
5	180.0315	25055	89.9	815	5793.8
6	181.0359	2451	8.8	75	572.7
7	192.0310	6401	23.0	218	1691.7
8	202.0127	3787	13.6	133	1135.6
9	224.0560	3583	12.9	155	1407.9
10	383.0511	4433	15.9	298	1677.7

Figure S39. The negative ion ESI mass spectrum of **7**.

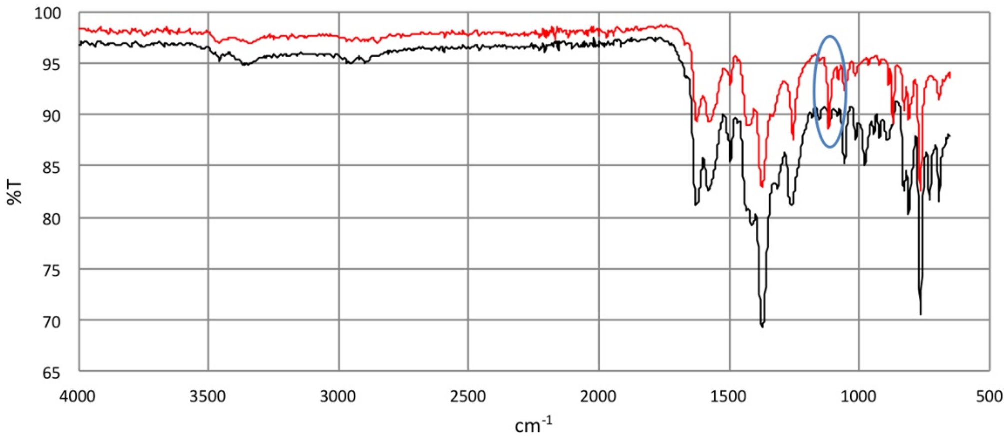

Figure S40. The FT-IR spectrum of **7** (red) in comparison to that of MIL-68(In)-NH_2 (black).

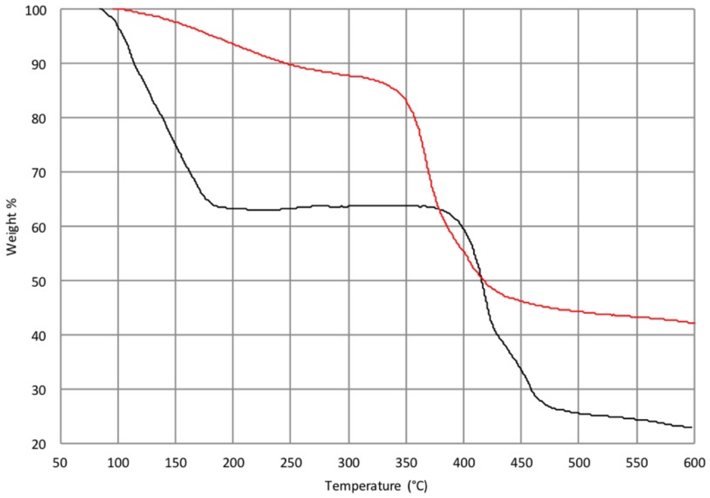

Figure S41. Thermogravimetric analyses for MIL-68(In)-NH_2 (black) and **7** (red).

8. Crystal structure determinations

Using the Olex2 interface,[S8] the structures were solved with ShelXS[S9] and refined using ShelXL.[S10]

8.1 Crystal structure of $[Zn_3(bdc\text{-}NH_2)_{1.32}(bdc\text{-}NHCH_2pyz)_{1.68}(dabco)]\cdot 2C_7H_8$ 5

Single crystal X-ray diffraction data for **5** were collected at 100 K on a Bruker Apex II diffractometer using synchrotron radiation (λ = 1.0333 Å) at Beamline 11.3.1 at the ALS Lawrence Berkeley National Laboratory. Key information about the data collection and structure refinement is given in Table S1.

Table S1. Data collection, structure solution and refinement for **5**.

Empirical formula	$C_{50.72}H_{49.72}N_{8.36}O_{12}Zn_3$
Formula weight	1164.50
Temperature/K	100.00(10)
Crystal system	trigonal
Space group	*R*–3*m*
a/Å	18.1826(7)
b/Å	18.1826(7)
c/Å	13.7312(6)
α/°	90
β/°	90
γ/°	120
Volume/Å^3	3931.4(3)
Z	2
ρ_{calc}/g cm^{-3}	0.984
μ/mm^{-1}	2.565
F(000)	1197.0
Crystal size/mm^3	0.03 × 0.03 × 0.02
2θ range for data collection/°	10.858 to 74.954
Index ranges	$-21 \le h \le 21$, $-21 \le k \le 21$, $-16 \le l \le 16$
Reflections collected	10674
Independent reflections	810 [R_{int} = 0.0490, R_{sigma} = 0.0236]
Data/restraints/parameters	810/31/84
Goodness-of-fit on F^2	1.222
Final *R* indexes [$I \ge 2\sigma(I)$]	R_1 = 0.0536, wR_2 = 0.1744
Final *R* indexes [all data]	R_1 = 0.0613, wR_2 = 0.1811
Largest diff. peak/hole / e Å^{-3}	0.76/–0.44

The asymmetric unit in this structure comprises one zinc centre (Zn1) with a site occupancy factor of 0.083333, a second zinc centre (Zn2) with an occupancy of 0.166667, a dabco nitrogen (N1) and carbon (C7) with site occupancies of 0.16667 and 0.5, respectively, plus a portion of a functionalised benzene dicarboxylate ligand which overall represents a mixture of bdc-NH_2 and bdc-$NHCH_2$pyz in a 34:56 ratio.

Atoms O1, O2, C1, C2, C3 and C4 from the dicarboxylate core are coincident with a space group mirror plane and, consequently all have half site-occupancy. This has chemical integrity in terms of a Zn:dianion:dabco ratio within the asymmetric unit of 0.25:0.25:0.16667, which equates to a ratio of 3:3:1 in the gross structure. Because of crystallographic symmetry, the functionalities in both bdc ligands are necessarily disordered. Hence, N2, which are common to both pendant groups has a site occupancy of 0.25. C5 and N3 were the only atoms that could be located with any reliability in the tag. Disorder and incomplete PSM conversion dictate that the site occupancy of these two fractional atoms is in the region of 0.14.

Crystallographic symmetry also means that the dabco CH_2 moieties are disordered. Given the tag disorder plus the fact that the electron density pertaining to the atoms therein becomes more diffuse with distance from the linker, the usual means of determining structural voids is not really applicable here. In fact, use of the PLATON squeeze algorithm, in this case, afforded a dataset against which refinement yielded higher residuals! However, TGA experiments indicated a mass loss that corresponded to a pair of toluene molecules for every three zinc centres present. Overall, this provides a formulation of $[Zn_3(bdc\text{-}NH_2)_{1.32}(bdc\text{-}NHCH_2pyz)_{1.68}(dabco)]\cdot 2C_7H_8$ for this compound. ADP restraints were added on merit for fractional occupancy atoms, in the final least-squares cycles to assist convergence. The asymmetric unit for **7** is shown in Figure S42 and the orientations adopted by the disordered pyrazole-containing substituent are shown in Figure S43.

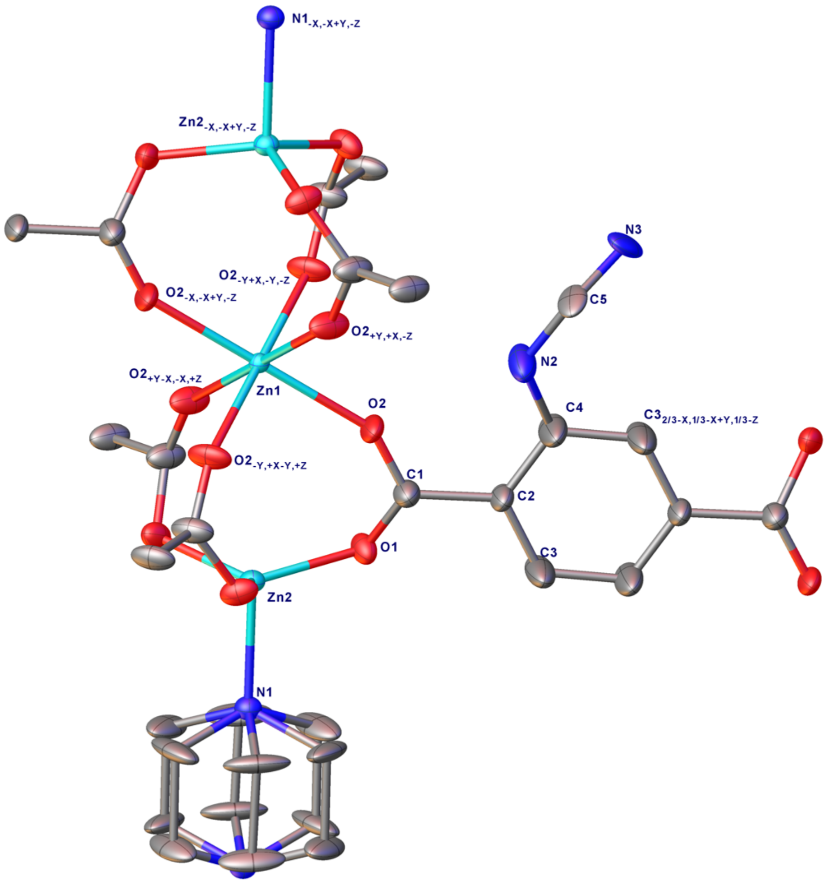

Figure S42. The asymmetric unit of $[Zn_3(bdc\text{-}NH_2)_{1.32}(bdc\text{-}NHCH_2pyz)_{1.68}(dabco)]\cdot 2C_7H_8$ **5**.

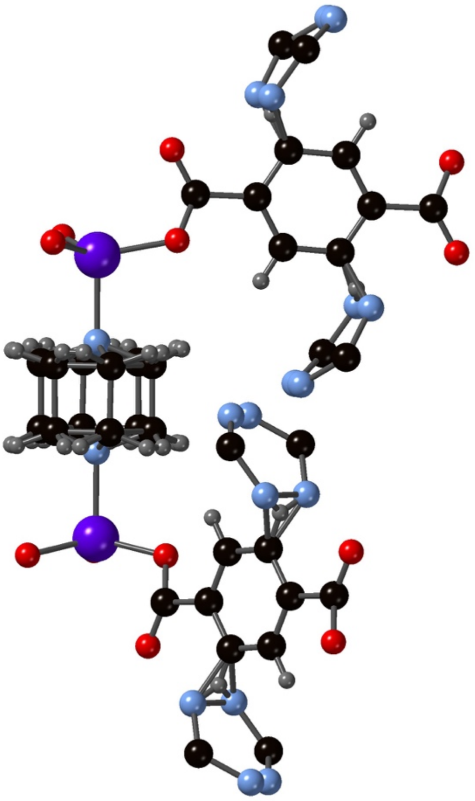

Figure S43. Part of the structure of $[Zn_3(bdc\text{-}NH_2)_{1.32}(bdc\text{-}NHCH_2pyz)_{1.68}(dabco)]\cdot 2C_7H_8$ **5**, showing the orientation of the pyrazole rings away from the bridging dabco ligands.

8.2 Crystal structure of $[In(OH)(bdc\text{-}NH_2)_{0.41}(bdc\text{-}NHCH_2OCH_3)_{0.30}(bdc\text{-}N{=}CH_2)_{0.29}]$ 7

Single crystal X-ray diffraction data for **7** were collected on an Agilent SuperNova diffractometer using CuKα radiation (λ = 1.5418 Å) at 150 K. Key information about the data collection and structure refinement is given in Table S2.

Table S2. Data collection, structure solution and refinement for **7**.

Empirical formula	$C_{12.09}InNO_{6.9}H_{13.6}$
Formula weight	398.14
Temperature/K	150.00(2)
Crystal system	orthorhombic
Space group	*Cmcm*
a/Å	21.7325(8)
b/Å	37.6102(14)
c/Å	7.22490(19)
α/°	90.0
β/°	90.0
γ/°	90.0
Volume/$Å^3$	5905.4(3)
Z	12
ρ_{calc}/g cm^{-3}	1.343
μ/mm^{-1}	9.828
F(000)	2368.0
Crystal size/mm^3	0.126 × 0.045 × 0.035
Radiation	CuKα (λ = 1.54184)
2θ range for data collection/°	8.136 to 139.974
Index ranges	$-13 \le h \le 26$, $-22 \le k \le 45$, $-8 \le l \le 8$
Reflections collected	3110
Independent reflections	3110 [R_{sigma} = 0.0779]
Data/restraints/parameters	3110/104/146
Goodness-of-fit on F^2	1.078
Final *R* indexes [$I \ge 2\sigma(I)$]	R_1 = 0.0401, wR_2 = 0.1087
Final *R* indexes [all data]	R_1 = 0.0548, wR_2 = 0.1197
Largest diff. peak/hole / e $Å^{-3}$	0.90/–0.93

Despite collection of a good data set for this structure, high symmetry in the diffraction pattern caused considerable difficulties in space group assignment. The credible contenders were the hexagonal $P6_3/mmc$ and the orthorhombic *Cmcm.* Both were explored in detail before presentation of the model here which is in the latter setting. A solution was brokered in the higher symmetry hexagonal option, but with 477 systematic absence violations, no detectable twinning options.

Space group *Cmcm* was then examined and a reasonable solution was extracted wherein the asymmetric unit was seen to comprise two metal centres with site occupancies of 0.5 and 0.25 for In1 and In2, respectively, half of a dicarboxylate ligand (based on O3) with C4, C5 C8 and C9 located at a crystallographic mirror plane, one quarter of a dicarboxylate ligand (based on O2) with C1 and C2 also located at a mirror plane. This latter moiety is also proximate to a second crystallographic mirror plane which contributes to generating the remainder. Two OH ligands (based on O1 and O5) with combined site occupancies of 0.75 are also present in the asymmetric unit and, finally, there was evidence for some diffuse solvent present in the framework. Initial refinement, pre PLATON SQUEEZE rendered an *R*1 value in the region of 9.6% - but with a poor weighting scheme.

PLATON analysis suggested that the data might have arisen from a 3-fold pseudo-merohedral twin, and a refinement against an appropriate dataset reduced the *R*1 value to the region of 6.5%. At this point, there was no evidence of the tag, so hydrogen atoms were attached to the aromatic carbons prior to implementation of PLATON SQUEEZE. Subsequent refinement against the arising dataset from this algorithm revealed electron density maxima in the region of the aromatic hydrogens, but at a longer distance from these carbons. This was interpreted as evidence for the nitrogen in the tags being disordered amongst the aryl carbons. Hence, the aromatic carbons were removed from the model, in favour of 3 isotropically refined, partial occupancy nitrogens per asymmetric unit. C–N distances were restrained to being similar given the low level of electron density at each site. 0.8 of a dioxane molecule per indium centre are included in the empirical formula given here based on TGA results for this material.

Atoms C3, C6 and C7 exhibited disorder which was modelled over 2 sites in 50:50, 66:34 and 66:34 ratios, respectively. Some ADP restraints were also included in the refinement to assist convergence. The asymmetric unit for **7** is shown in Figure S44.

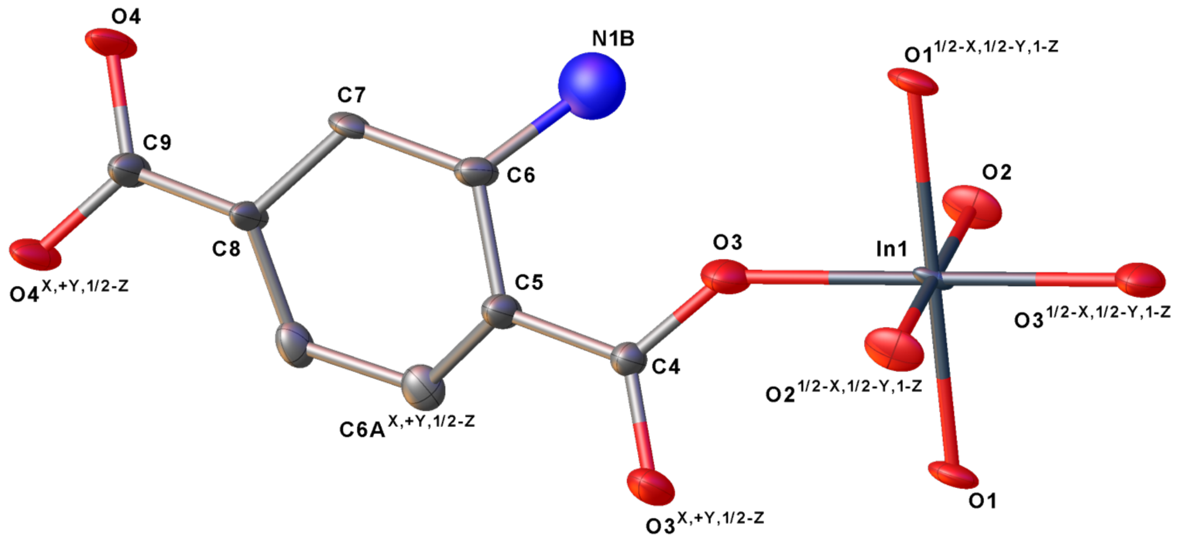

Figure S44. The asymmetric unit of $[In(OH)(bdc\text{-}NH_2)_{0.41}(bdc\text{-}NHCH_2OCH_3)_{0.30}(bdc\text{-}N{=}CH_2)_{0.29}]$ **7**.

9. References

S1. J. H. Cavka, S. Jakobsen, U. Olsbye, N. Guillou, C. Lamberti, S. Bordiga and K. P. Lillerud, *J. Am. Chem. Soc.* 2008, **130**, 13850.
S2. M. Eddaoudi, J. Kim, N. Rosi, D. Vodak, J. Wachter, M. O'Keeffe and O. M. Yaghi, *Science* 2002, **295**, 469.
S3. H. Chun, D. N. Dybtsev, H. Kim and K. Kim, *Chem. Eur. J.* 2005, **11**, 3521
S4. C. Volkringer, M. Meddouri, T. Loiseau, N. Guillou, J. Marrot, G. Férey, M. Haouas, F. Taulelle, N. Audebrand and M. Latroche, *Inorg. Chem.*, 2008, **47**, 11892.
S5. S. J. Garibay and S. M. Cohen, *Chem. Commun.*, 2010, **46**, 7700.
S6. H. Yim, E. Kang and J. Kim, *Bull. Korean Chem. Soc.*, 2010, **31**, 1041.
S7. D. N. Dybtsev, H. Chun and K. Kim, *Angew. Chem. Int. Ed.*, 2004, **43**, 5033.
S8. O. V. Dolomanov, L. J. Bourhis, R. J. Gildea, J. A. K. Howard and H. Puschmann*, J. Appl. Cryst.*, 2009, **42**, 339.
S9. G. M. Sheldrick, *Acta Crystallogr. Sect. A*, 2008, **64**, 112.
S10. G. M. Sheldrick, *Acta Crystallogr. Sect. C*, 2015, **71**, 3.
